# Environmental analysis of plasma-based nitrogen fixation powered by renewable energy

**DOI:** 10.1039/d6gc04056j

**Published:** 2026-10-20

**Authors:** Jose Osorio-Tejada, Yury Gorbanev, Vikram Kenjle, Annemie Bogaerts, Volker Hessel

**Affiliations:** a School of Engineering, University of Warwick UK jose.osorio-tejada@warwick.ac.uk; b Research Group Energy and CO_2_, Department of Mechanical Engineering Universidad de Zaragoza Spain; c Research group PLASMANT and PLASMA Center of Excellence, Department of Chemistry, University of Antwerp Belgium annemie.bogaerts@uantwerpen.be; d Electrification Institute, University of Antwerp Belgium; e School of Chemical Engineering, Adelaide University Australia

## Abstract

This study assesses the environmental performance of plasma-based nitric acid (HNO_3_) production powered by renewable electricity using process modelling and comparative life cycle assessment. Two plasma technologies, a gliding arc plasmatron (GAP) and a rotating gliding arc (RGA), were evaluated under air-fed (N_2_ : O_2_ 4 : 1) and oxygen-enriched (N_2_ : O_2_ 1 : 1) configurations using experimental reactor data and real photovoltaic (PV) generation profiles, and were benchmarked against the conventional steam methane reforming–Haber–Bosch–Ostwald process. At atmospheric pressure and an annual electricity mix of 41.5% solar, plasma-based routes show global warming impacts of 1.82–2.25 kg CO_2_ eq. per kg HNO_3_, while the conventional route emits 1.93 kg CO_2_ eq. per kg HNO_3_ at the factory gate. When the solar share is over 60%, all plasma plant configurations emit lower carbon than the benchmark, reaching emissions as low as 0.99 kg CO_2_ eq. per kg HNO_3_ for the air-fed GAP reactor when the plant is powered 100% by solar PV. The results were also highly sensitive to future improvements in plasma electricity demand: a scenario reducing demand by 20% decreased climate change impacts by approximately 11–18%, with all four plasma configurations achieving lower impacts than the benchmark. Similar benefits were observed for ozone formation and terrestrial acidification. Seasonal variation in solar/grid share (18–64%) changed global warming impacts by approximately 43%. Plasma routes also showed higher toxicity and land use impacts, mainly due to electrical infrastructure and PV systems. Oxygen enrichment improved NO_*x*_ yields but required additional energy for O_2_ production; it became environmentally favourable only below approximately 0.5 kWh kg^−1^ O_2_. Elevated-pressure RGA operation at 4 bar substantially improved performance, reducing total plant energy cost by 43% and 27% for the 4 : 1 and 1 : 1 configurations, respectively, and lowering global warming impacts to 1.19 and 1.10 kg CO_2_ eq. per kg HNO_3_. Overall, plasma-based HNO_3_ production shows strong low-carbon potential, particularly under elevated-pressure operation.

## Abstract 2

Gliding arc plasmas (GAP/RGA) enable efficient NO_*x*_ synthesis. Life cycle assessment reveals environmentally competitive local HNO_3_ production, with impacts heavily dictated by capacity factors and solar/grid share.

## Graphical abstract

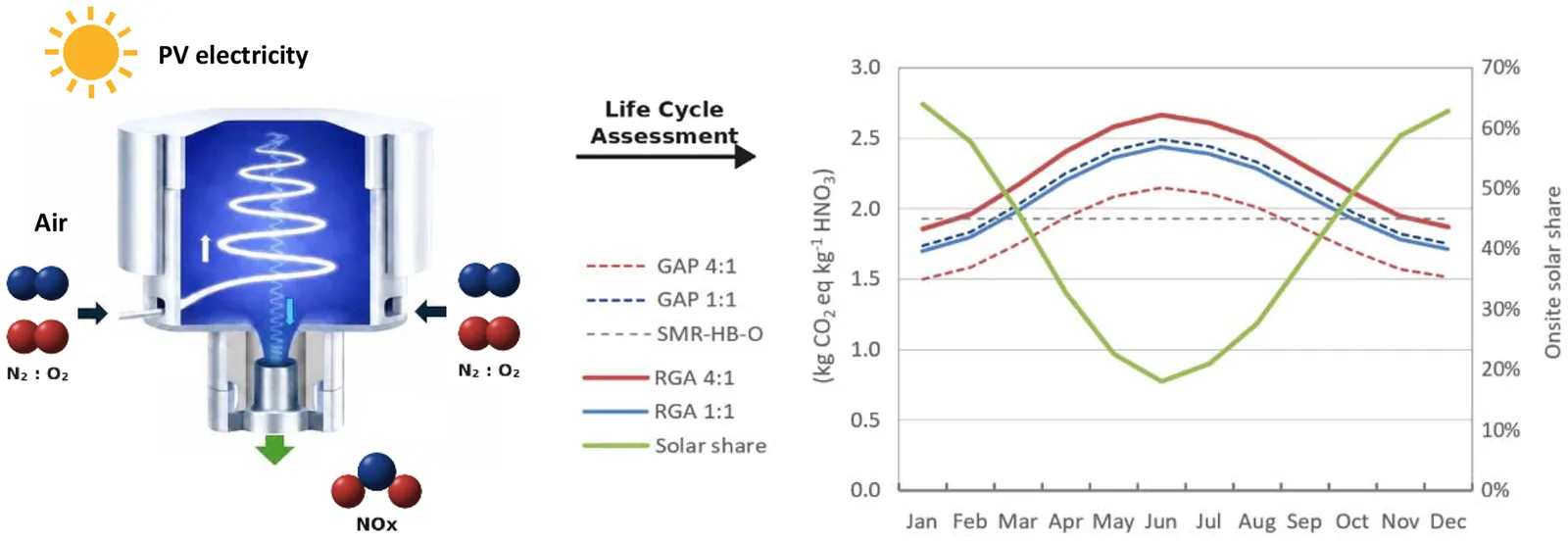

**Green foundation**

1. This work advances green chemistry by providing the first comprehensive life cycle assessment of renewable-electricity-driven plasma nitric acid production using experimental reactor data, process modelling, and realistic renewable-energy operation. It establishes a robust framework for evaluating electrified nitrogen fixation beyond energy efficiency alone.

2. Under realistic solar-powered operation, emissions fall as low as 0.99 kg CO_2_ eq. per kg HNO_3_ once the on-site solar share exceeds 60%, compared with 1.93 for the conventional route. Operating the plasma reactor at elevated pressure (4 bar) further improves performance, cutting energy demand by up to 43%.

3. Further advances require improved plasma energy efficiency, low-energy oxygen generation, and greater integration with renewable electricity and energy storage to minimise remaining impacts, particularly those associated with electricity demand, toxicity, and land use.

## 1 Introduction

In recent years, particularly following the adoption of the United Nations Sustainable Development Goals, the development of sustainable chemical manufacturing has become increasingly important for addressing global environmental challenges.^1^ Nitric acid (HNO_3_) is a key industrial chemical, widely used in fertilisers and explosives.^2^ Its conventional production relies on ammonia (NH_3_) synthesised *via* the Haber–Bosch (HB) process, which accounts for ∼90% of global NH_3_ production and depends heavily on fossil fuels for both energy and hydrogen supply.^3–5^ Combined with the Ostwald process towards HNO_3_, this HB–Ostwald (HB–O) route is energy-intensive, infrastructure-heavy, and poorly suited for decentralised or renewable-based production.

Hydrogen for the HB process is typically produced from natural gas *via* steam methane (CH_4_ + H_2_O into CO_*x*_ and H_2_) reforming, followed by NH_3_ synthesis from N_2_ and H_2_. In the Ostwald process, NH_3_ is oxidised to NO, further converted to NO_2_, and absorbed in water to form HNO_3_. Most energy cost occurs in the HB stage, primarily due to hydrogen production and high-pressure ammonia synthesis.^6^ Despite efficiency improvements since its development, the process still requires ∼28 MJ kg^−1^ NH_3_ and accounts for about 1.8% of global energy cost, highlighting its misalignment with sustainability goals.^7^

Because currently HNO_3_ production depends directly on NH_3_ synthesis, nearly half of the world's population relies indirectly on the HB process.^8^ This has driven interest in alternative nitrogen fixation routes, including enzymatic, plasma-based, and (electro)catalytic methods.^9,10^ Among these, plasma-based fixation is particularly attractive due to its flexibility and compatibility with renewable electricity.^10–17^

In plasma systems, NO_*x*_ can be produced from N_2_ : O_2_ mixtures, including air, or from N_2_/H_2_O-containing feeds.^18,19^ The concept dates back to the Birkeland–Eyde process, which used thermal arcs to convert air into NO_*x*_, later absorbed into HNO_3_.^20^ Although replaced by the HB–Ostwald process due to lower energy cost,^15^ modern research revisits plasma technologies as potential alternatives. Often, the most energy-efficient NO_*x*_ formation pathway is associated with the non-thermal Zeldovich mechanism, involving N_2_ and O_2_ vibrational excitation followed by vibrationally-enhanced reaction pathways leading to NO and NO_2_.^12,18,19,21–23^ However, most recent research developments show that the best metrics are achieved with “warm” plasmas (*e.g.* microwave plasma, plasma arcs, *etc.*), which have lower degrees of non-equilibrium.^18,24–26^ Recent findings,^27^ supported by fully coupled, predictive computational fluid dynamics modelling,^28^ indicate that the thermal mechanism plays the dominant role.

Furthermore, the minimum energy cost (EC) for NO production in atmospheric-pressure air plasma at vibrational–translational (VT) non-equilibrium has recently been revised,^29^ from the exceptionally low EC of 0.2 MJ mol^−1^ at low pressure, predicted by Rusanov *et al.*, (1981),^30^ for the vibrationally-enhanced Zeldovich mechanism, to about 1.4–1.8 MJ mol^−1^ (depending on the vibrational distribution function). In parallel, NO formation due to chemical non-equilibrium but (VT) thermal equilibrium was recently proposed as a new pathway toward optimising plasma-based NO production, also yielding an EC of 1.8 MJ mol^−1^, and the authors stated that this value may be further reduced by implementing heat recovery, because a lot of energy is now wasted as heat losses to the environment.^29^

Overall, while comparisons with the HB process often focus on EC and product concentration, plasma-based systems differ fundamentally, offering advantages such as electrification, modularity, and suitability for decentralised operation.

Environmental assessments also highlight this potential. A life cycle assessment (LCA) by Anastasopoulou *et al.* (2016)^31^ suggested plasma-based HNO_3_ production could become competitive under conditions such as renewable electricity use, improved NO concentrations (≥10%), and the implementation of energy recovery or tail-gas recycling. However, their analysis was based on an EC of 0.84 MJ mol^−1^ NO_*x*_, derived from a low-pressure (40 mbar) microwave plasma study^32^ a value that has not been reproduced in subsequent decades; see also the discussion above about more realistic values of EC. Moreover, low-pressure plasma systems present significant scalability challenges, as they require complex vacuum infrastructure and are difficult to operate continuously at a large scale. Consequently, atmospheric-pressure plasma processes are now preferred for industrial applications.^15–17,29,33^

Unlike plasma systems, the HB process is difficult to scale down due to its large production capacity (≥100 t NH_3_ per day) and operation under high temperature and pressure (350–500 °C, 100–300 bar), limiting its compatibility with intermittent renewable energy.^7^ Emerging alternatives include milder thermo-catalytic and electrochemical NH_3_ synthesis routes.^34,35^

With the increasing availability of low-cost renewable electricity, plasma technology offers advantages such as rapid start-up, operational flexibility, and suitability for small-scale applications.^11,12,36^ However, previous environmental studies often rely on simplified assumptions, particularly regarding plant capacity factors. The capacity factor, defined as the ratio of the total operating hours to the total hours in a year, may be significantly reduced when using intermittent energy sources.^14,15,37^

Building upon our group's previous techno-economic and LCA frameworks for plasma-assisted chemical processes *via* CO_2_ splitting^38,39^ and syngas production,^39^ this work expands the paradigm to plasma-driven nitric acid production. While our prior studies established foundational approaches for assessing plasma system impacts, critical gaps remain regarding full-scale plant integration and realistic operational profiles for nitrogen fixation. Here, we advance previous methodologies by incorporating full-plant Aspen Plus process simulations,^40^ evaluating location-specific solar photovoltaic (PV) generation profiles with dynamic capacity factor modelling, and systematically investigating process parameters unique to NO_*x*_ synthesis, including system pressurisation and oxygen-enrichment strategies.

In this context, this study provides a comprehensive environmental assessment of plasma-based nitric acid production. It evaluates different N_2_ : O_2_ feed compositions under realistic energy-supply conditions, combining experimental data from two gliding arc plasmas, namely a gliding arc plasmatron (GAP) and a rotating gliding arc (RGA) reactor, with operational data from a solar PV facility at the Adelaide University campus in Roseworthy, South Australia.

## 2 Methods

### 2.1 Process design

Arc plasmas are among the most studied types of plasma historically applied to nitrogen fixation into NO_*x*_ since the Birkeland–Eyde process,^41^ with a specific interest in gliding arc (GA) plasma reactors.^18,26,41^ A reverse vortex GAP was recently demonstrated to be an efficient plasma reactor for a lab-scale plasma-based gas conversion processes such as CO_2_ plasmalysis,^42^ as well as dry reforming of methane^43^ and NH_3_ cracking.^44^ Moreover, a GAP with post-plasma carbon bed demonstrated drastic performance improvements for CO_2_ splitting.^45,46^ Finally, a GAP-based multi-reactor was used in a study on upscaling arc plasma CO_2_ splitting.^47^

In our earlier work, we used this GAP reactor to produce NO_*x*_ (NO and NO_2_) at slightly elevated pressure (1.25 atm) and 10 L min^−1^ feed gas flow, as described in Vervloessem *et al.*, (2020).^19^ In these experiments, the plasma arc was ignited in a DC discharge mode, as is common for arc research.^27^ Interestingly, the concentration of produced NO_*x*_ (1.49 vol%) did not increase when switching from air (N_2_ : O_2_ 4 : 1) to oxygen-enriched air (N_2_ : O_2_ 1 : 1). This was counter-intuitive: a trend observed in almost all cases of N_2_ oxidation by O_2_ shows that NO_*x*_ production increases with the increase of oxygen fraction.^48^ In turn, the observed increase in power at higher oxygen content resulted in higher EC values, which increased from 3.62 MJ mol^−1^ N (for N_2_ : O_2_ 4 : 1) to 4.09 MJ mol^−1^ N (1 : 1). In any case, the EC was above the state-of-the-art and the thermodynamic limit,^27,29,49^ although elevated pressure plasma is known to improve the process metrics.^41^

In contrast, our new results obtained with an AC discharge in the same GAP reactor were in good agreement with the trends reported in literature. Specifically, for the N_2_ : O_2_ ratios of 4 : 1 and 1 : 1, the NO_*x*_ concentrations were 1.20 vol% and 1.55 vol%, respectively. The corresponding EC was *ca.* 2.7 and 2.4 MJ mol^−1^ N. These values fall within the typical results obtained with arc plasmas.^11,24^ Furthermore, they were obtained under atmospheric pressure (*i.e.*, 1 atm), the same way as most reported research on arc plasmas.^48^ Hence, we used these data in our present work as representative of the state-of-the-art median.

The schematic representation of the GAP plasma and the process diagram are shown in Fig. 1(a) and (b), respectively.

**Fig. 1:**
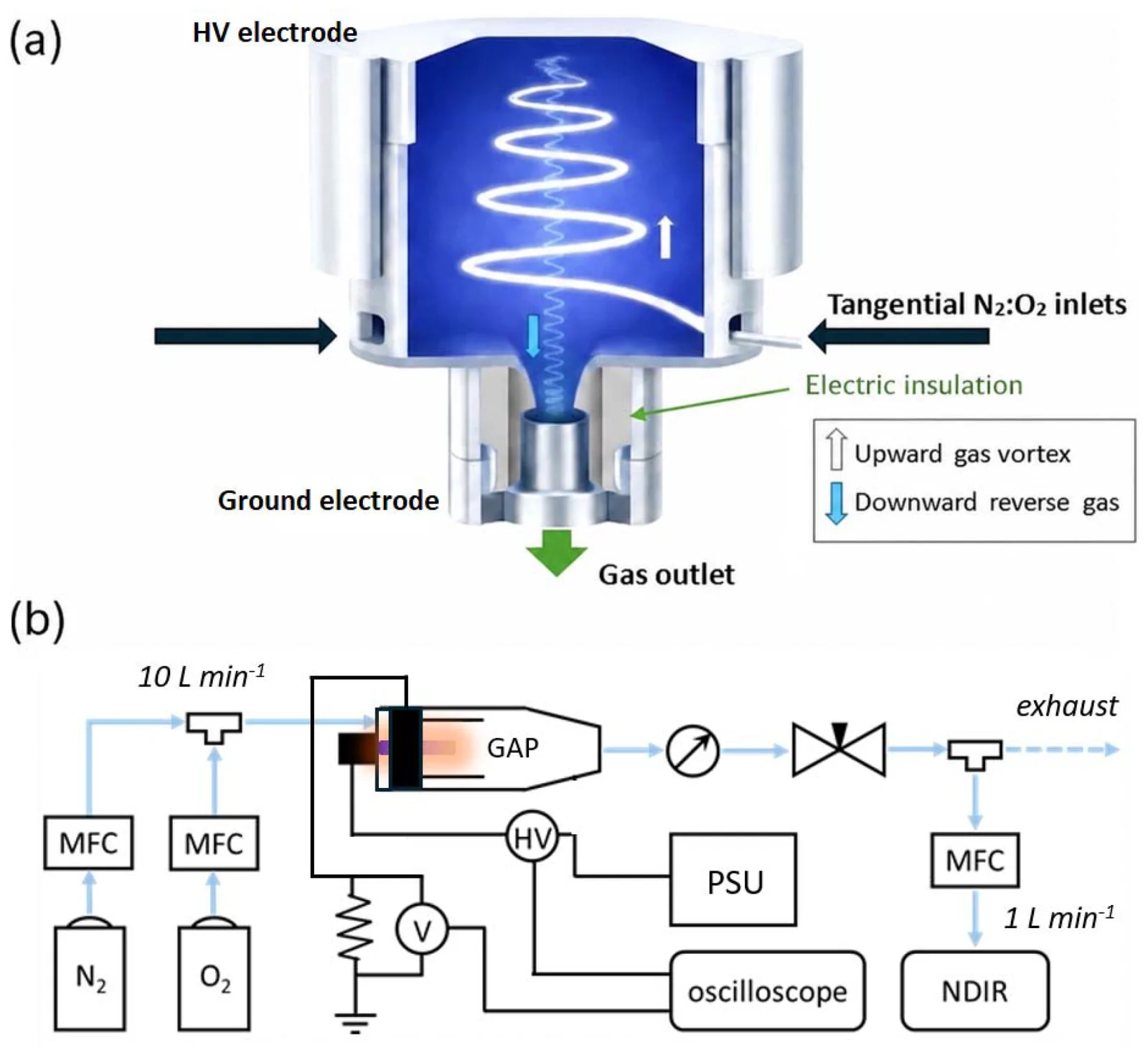
GAP reactor with forward and reverse vortex flow (a), and process diagram of NO_*x*_ production with the GAP plasma setup (b).

The feed gas flow (N_2_ : O_2_ of 4 : 1 or 1 : 1, total flow rate 10 L min^−1^) was supplied using mass flow controllers (MFC; Bronkhorst) connected to N_2_ and O_2_ gas cylinders (both 99.999 vol%, Praxair). The feed gases N_2_ and O_2_ were mixed using a T-connector, and introduced into the GAP plasma reactor (Fig. 1b). The reactor comprised the main part (electrode system with ceramic separators; Fig. 1a) and the long reactor body (stainless steel; not shown in Fig. 1), with the detailed description found elsewhere.^46^ The discharge was driven by a high-voltage AC power supply unit (PSU; AFS GmbH G10P). The electrical characteristics were monitored with a digital oscilloscope (Keysight InfiniiVision DSOX1102A) using a high-voltage probe (Tektronix P6015A). The voltage over a shunt resistor was measured to estimate the current values. The total power consumption (defined as plug power) was measured using a power meter (Voltcraft). Plasma power was calculated as an average of instantaneous plasma power values (voltage multiplied by current). The exhaust gas flow containing NO_*x*_ was split with T-connector, and a small fraction of it (*ca.* 1 L min^−1^) was directed towards an analyser with an MFC. The concentration of the produced NO_*x*_ in the gas outlet from the GAP reactor was analysed with a non-dispersive infrared analyser (NDIR; Emerson) equipped with NO, NO_2_, and N_2_O NDIR sensors. No N_2_O was detected in the experiments. This work considered actual parameter values for different N_2_ : O_2_ gas feeds, such as plasma power, NO and NO_2_ concentrations, production rates, and the nitrogen fixation energy costs based on plasma-deposited power (as typically reported in literature, “EC plasma”) as well as on total energy consumption drawn from the mains (for a proper feasibility representation, “EC plug”). EC plasma and EC plug were calculated based on the measured voltage and current, and on the measured plug power, respectively, as detailed in Tsonev *et al.*, (2023).^41^ Plug-to-plasma energy efficiency values are calculated as a fraction of total consumed power from the mains that is deposited into the plasma. All parameters are listed in Table 1.

**Table 1:** Operating parameters of the GAP (AC PSU) and RGA (DC PSU) setups for NO_*x*_ production at atmospheric pressure

Type of reactor	N_2_ : O_2_ feed ratio	N_2_ : O_2_ feed flow rate	Plasma power	EC plasma	EC plug	NO_*x*_ concentration (vol%)			Production rate		Plug-to-plasma efficiency (%)
		(L min^−1^)	(W)	(MJ mol^−1^ NO_*x*_)	(MJ mol^−1^ NO_*x*_)	NO	NO_2_	Total	(L min^−1^)	(g h^−1^)	
GAP	4 : 1	10	241.1	2.7	4.9	0.66	0.54	1.20	0.120	11.93	55
	1 : 1	10	276.8	2.4	4.7	0.69	0.86	1.55	0.154	16.08	51
RGA^a^	4 : 1	12	495.5	4.2	12.1	0.96	0.37	1.33	0.159	14.70	34
	1 : 1	12	698.8	3.3	7.3	1.12	1.28	2.40	0.286	29.54	45

a Data from Tsonev *et al.* (2023).^41^

In addition, Table 1 also contains data for another GA plasma, namely a rotating gliding arc (RGA) plasma, operated in DC discharge mode, used for nitrogen fixation into NO_*x*_.^41^ Our previous works with RGA demonstrated very good metrics for nitrogen oxidation,^18,26,41^ although the EC was worse than with the GAP results reported here at atmospheric pressure and higher throughput, *i.e.* ≥10 L min^−1^, but the NO_*x*_ concentration was generally better (see Table 1). Indeed, adding them allows a more balanced overview of various lab-scale arc plasmas at the state-of-the-art. The RGA plasma was driven by a DC PSU (Technix SR12KV-10KW). The NO_*x*_ and power measurements were conducted the same way as for GAP, with the Emerson NDIR gas analyser and a high voltage probe (Tektronix P6015A) connected to a digital oscilloscope (Keysight InfiniiVision DSOX1102A), respectively. All further experimental details on the RGA nitrogen fixation experiments are found elsewhere.^41^

The study evaluated N_2_ : O_2_ feed ratios of 4 : 1 (air-like) and 1 : 1 to assess plasma performance under different gas compositions. While 4 : 1 reflects standard operating conditions, the 1 : 1 ratio was included based on literature reporting improved reaction efficiency, higher NO_*x*_ yields, and reduced energy cost.^25^ This comparison allows assessment of whether the benefits of O_2_ enrichment justify its associated energy and environmental costs.

Plug-to-plasma efficiency was calculated for the GAP reactor to capture system-level performance. Earlier studies focused primarily on reactor-level energy consumption to investigate fundamental plasma behaviour.^38^ However, recent work has shifted towards evaluating overall system efficiency, particularly in the context of industrial scalability, motivating the inclusion of plug-to-plasma metrics in this study. The experimental setup reports energy measured at the plug, thus accounting for losses in the power supply unit, resulting in efficiencies of approximately 51–55% (see Table 1). These values correspond to a laboratory-scale setup using a multipurpose PSU, which is not optimised for a specific reactor configuration. In industrial applications, dedicated PSUs are typically designed to match the plasma reactor, resulting in significantly higher efficiencies. Literature reports plug-to-plasma efficiencies up to 92% for optimised large-scale systems,^47,50^ with values of 80% achievable on a semi-pilot scale as demonstrated by our previous works with GAP plasma reactors.^47,51,52^ Therefore, we assume a value of 80% in this study as a realistic estimate for scaled-up GAP and RGA systems.

The SMR–HB–Ostwald process remains the industrial benchmark for nitric acid production (Fig. 2a). Methane is first reformed with steam (SMR) to produce syngas (H_2_ and CO), followed by secondary reforming with air, which supplies heat *via* partial oxidation and introduces N_2_. The gas mixture then undergoes water–gas shift (WGS) to increase H_2_ yield. Subsequent gas purification removes CO_2_ (*via* scrubbing) and residual CO (*via* methanation), ensuring a clean feed for ammonia synthesis. The purified H_2_ and N_2_ are converted to NH_3_ in the HB loop under high pressure (100–300 bar) and moderate temperatures (400–500 °C).^53^

**Fig. 2:**
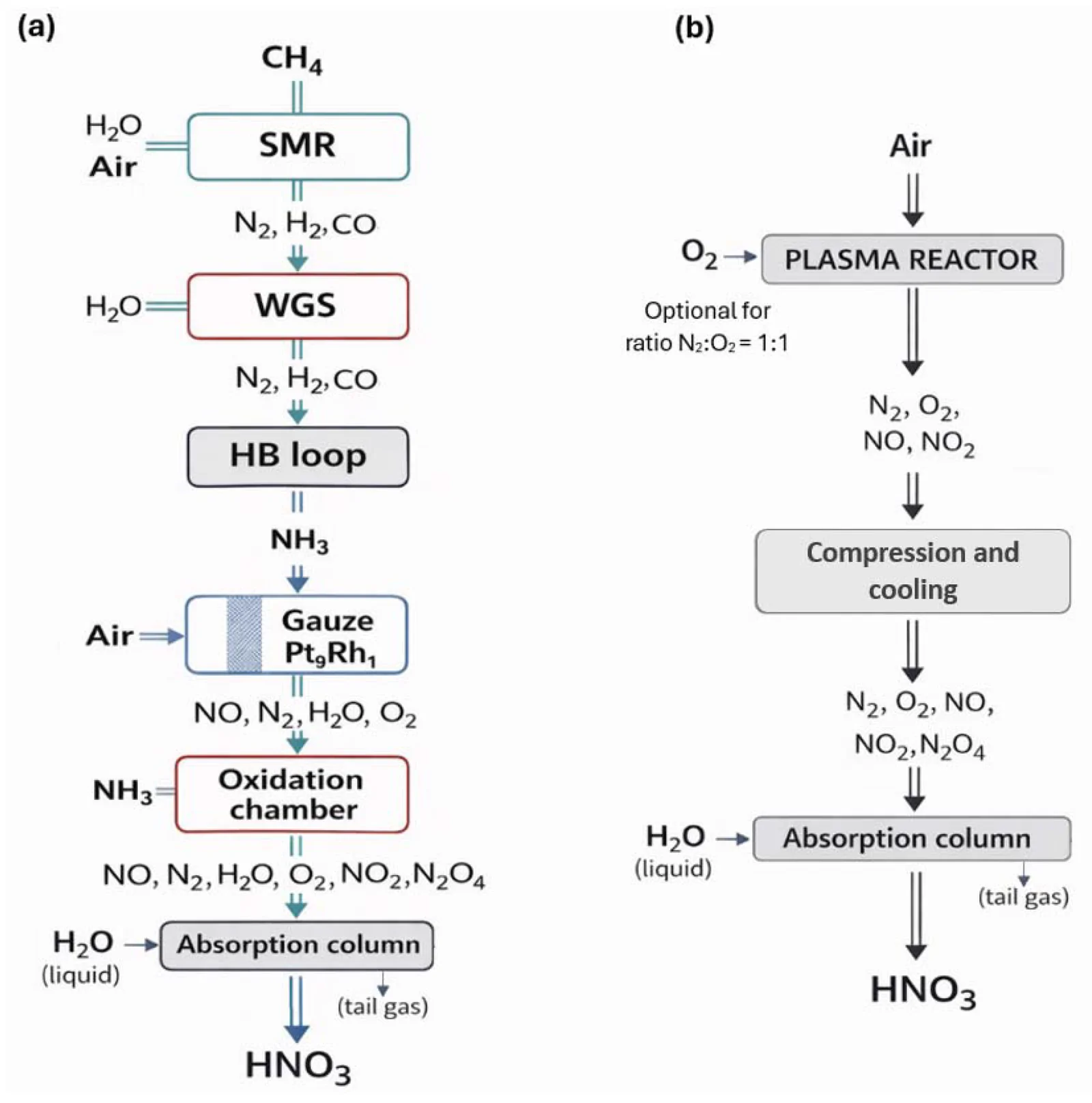
HNO_3_ production by (a) the conventional SMR–HB–Ostwald process and (b) the plasma-based process (see detailed description in text).

The produced NH_3_ is oxidised in the Ostwald process over a Pt–Rh gauze catalyst (700–950 °C, 4–10 bar), forming NO, which is further oxidised to NO_2_. The resulting gases are absorbed in water to produce HNO_3_, while tail gases are treated to meet emission standards. Although high NH_3_ conversion efficiencies (≥96%) are achieved, the process is capital-intensive and affected by catalyst degradation and Pt losses.^54^

In contrast, plasma-based systems enable direct conversion of N_2_ and O_2_ into NO_*x*_ at near-atmospheric pressure using electricity. The process comprises three main units (Fig. 2b): (i) a plasma reactor generating NO and NO_2_, (ii) a compression and cooling stage promoting NO_2_ formation and dimerisation into N_2_O_4_, and (iii) an absorption unit converting NO_2_ and N_2_O_4_ into HNO_3_.

A nitric acid concentration of 60 wt% was selected as the functional unit for the plasma-based system, as it represents a typical industrial concentration for nitric acid production and facilitates comparison with conventional benchmarks reported in the literature.^55–57^

### 2.2 Renewable energy supply

The solar farm is located at the Roseworthy Campus of Adelaide University, Australia, with an installed capacity of 1204 kW-peak (kWp), comprising 3168 monocrystalline silicon PV modules (JA Solar JAM72S01-380/PR/1500 V, 380 Wp each) arranged in two sub-arrays with a combined module area of 6153 m^2^, and a battery system with a total storage capacity of 1550 kWh. Full array specifications are provided in SI Table S2. Photographs of the solar farm are presented in Fig. 3, and the corresponding site layout, illustrating the scale of the electrical balance-of-system infrastructure relative to the full PV array, is presented in Fig. 4.

**Fig. 3:**
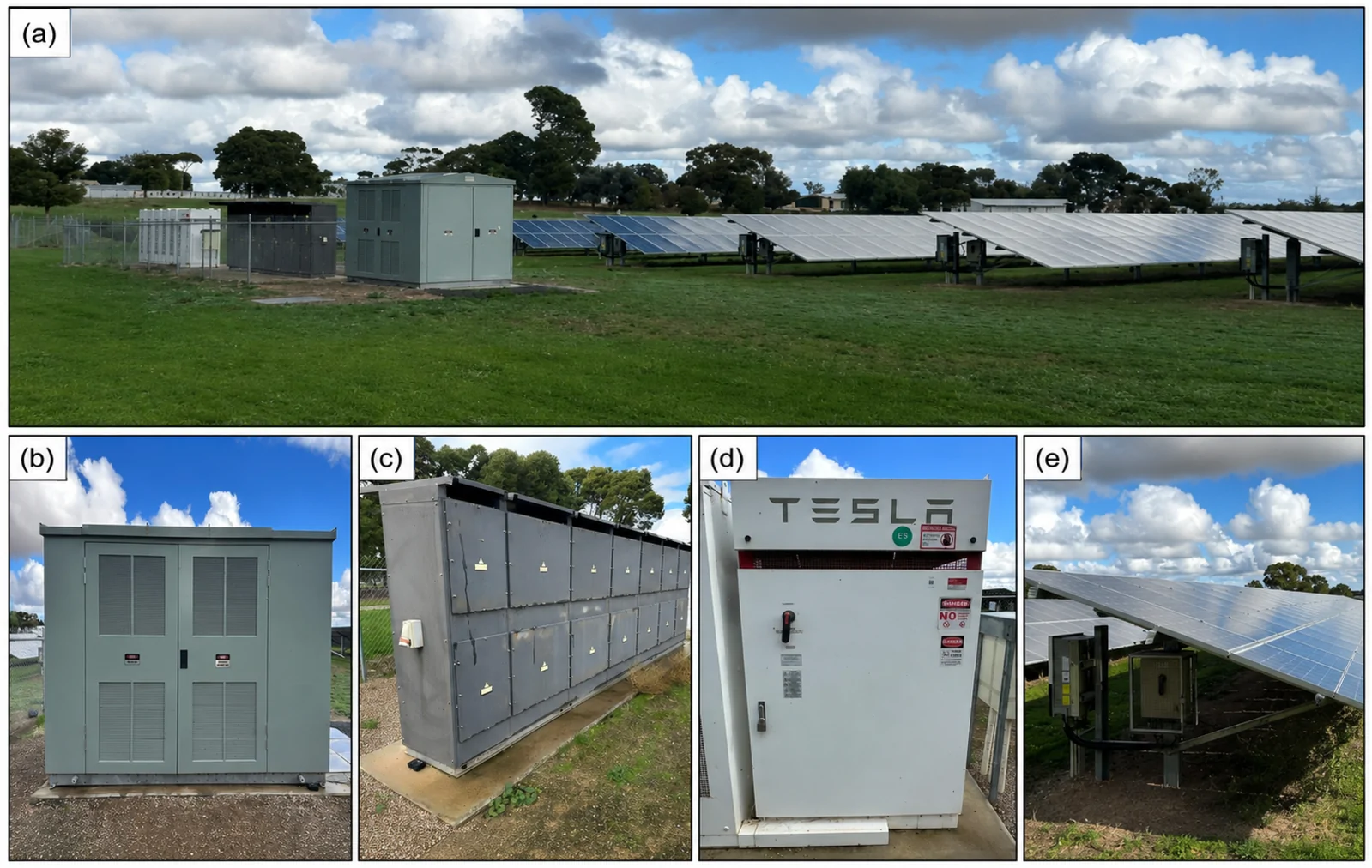
Photographs of the Roseworthy campus solar farm and associated energy infrastructure: (a) general overview of the 1.2 MW ground-mounted PV system and electrical equipment; (b) 400 V/2500 A PV distribution board; (c) 11 kV high-voltage step-up transformer; (d) Tesla battery energy storage system (BESS) powerpack unit; and (e) segment of the fixed-tilt monocrystalline solar PV array, oriented northward. Full descriptions in Table S1 of SI.

**Fig. 4:**
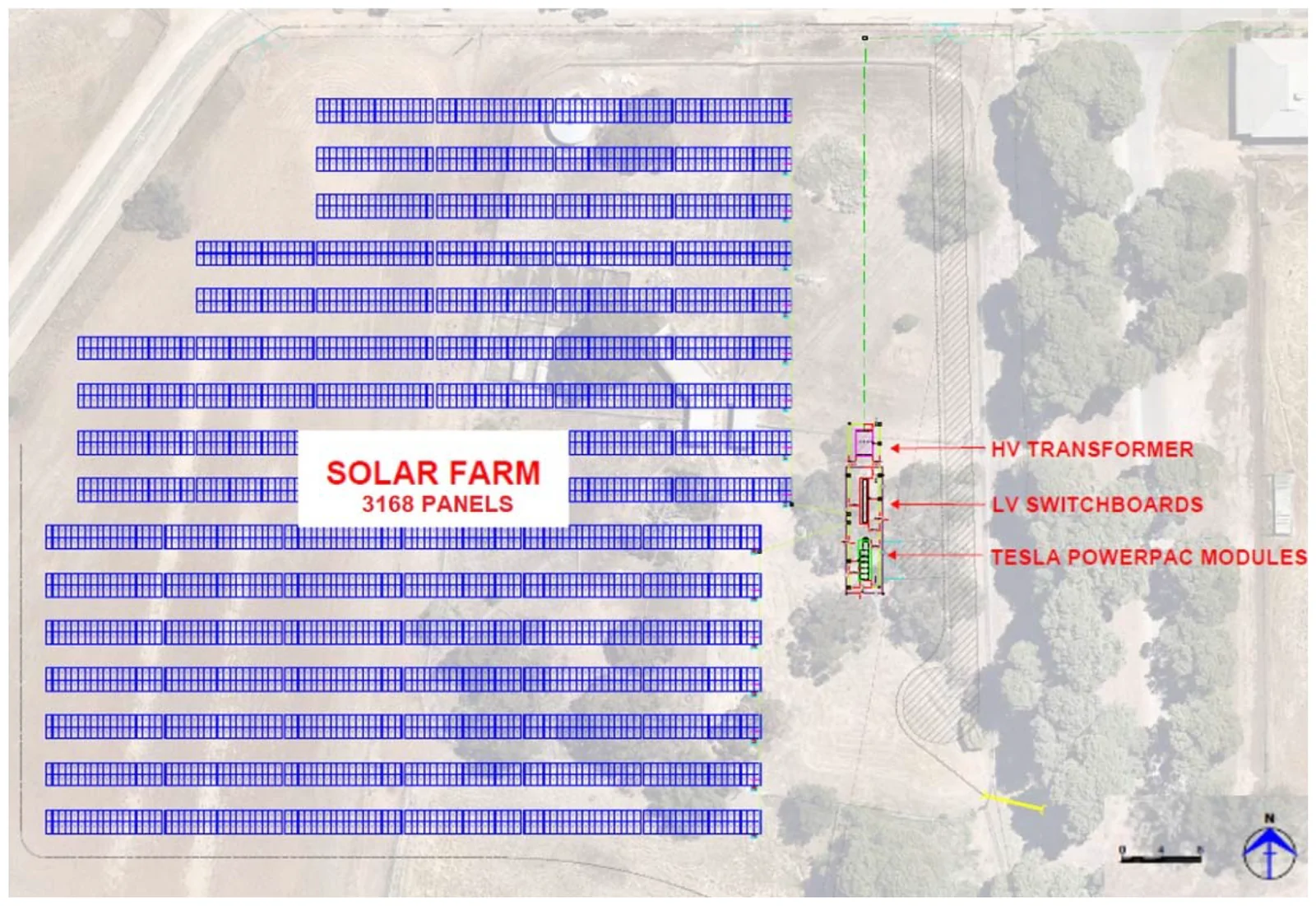
Site layout of the Roseworthy solar farm, showing the photovoltaic array footprint and the locations of the HV transformer, LV switchboards, and battery storage system. Drawing No. J2240-372. Prepared by Athavan Ravindra, Clean Technology Partners (Australia).

Although the existing solar farm currently supplies campus loads, this case study assumes the facility is dedicated exclusively to the proposed nitric acid plant. Consequently, its total electricity generation and associated environmental impacts are fully allocated to nitric acid production.

The estimation of the solar farm's energy production was carried out using solar radiation data from the NASA Surface Meteorology and Solar Energy dataset, as implemented in the Hybrid Optimisation Model of Electric Renewables (HOMER) software. Additionally, measured operational data from the solar farm over the past year were analysed. These data include half-hourly electricity generation over a full calendar year and were used to support a detailed plant dispatch analysis.

### 2.3 Life cycle assessment

LCA was performed for each plasma feed ratio to evaluate the environmental performance of HNO_3_ production *via* plasma-based nitrogen fixation. The analysis follows the methodological framework established in ISO 14044,^58^ and was implemented using the SimaPro software v9, an LCA software used to model product and process life cycles and quantify their associated environmental impacts, with background data obtained from the Ecoinvent 3.8 life cycle inventory database, which provides datasets for materials, energy, transport, and other background processes.^59^ This LCA supports a comparative sustainability assessment of decentralised plasma-based processes *versus* the conventional pathway.

The LCA aimed to assess the cradle-to-gate environmental impacts of producing 1 tonne of 60 wt% nitric acid *via* plasma–assisted nitrogen fixation. The system boundaries include all activities from ambient air capture and compression, plasma NO/NO_2_ formation, cooling, and NO_2_ absorption into water to produce HNO_3_, as illustrated in Fig. 5. Auxiliary units for tail gas treatment and energy recovery are also included.

**Fig. 5:**
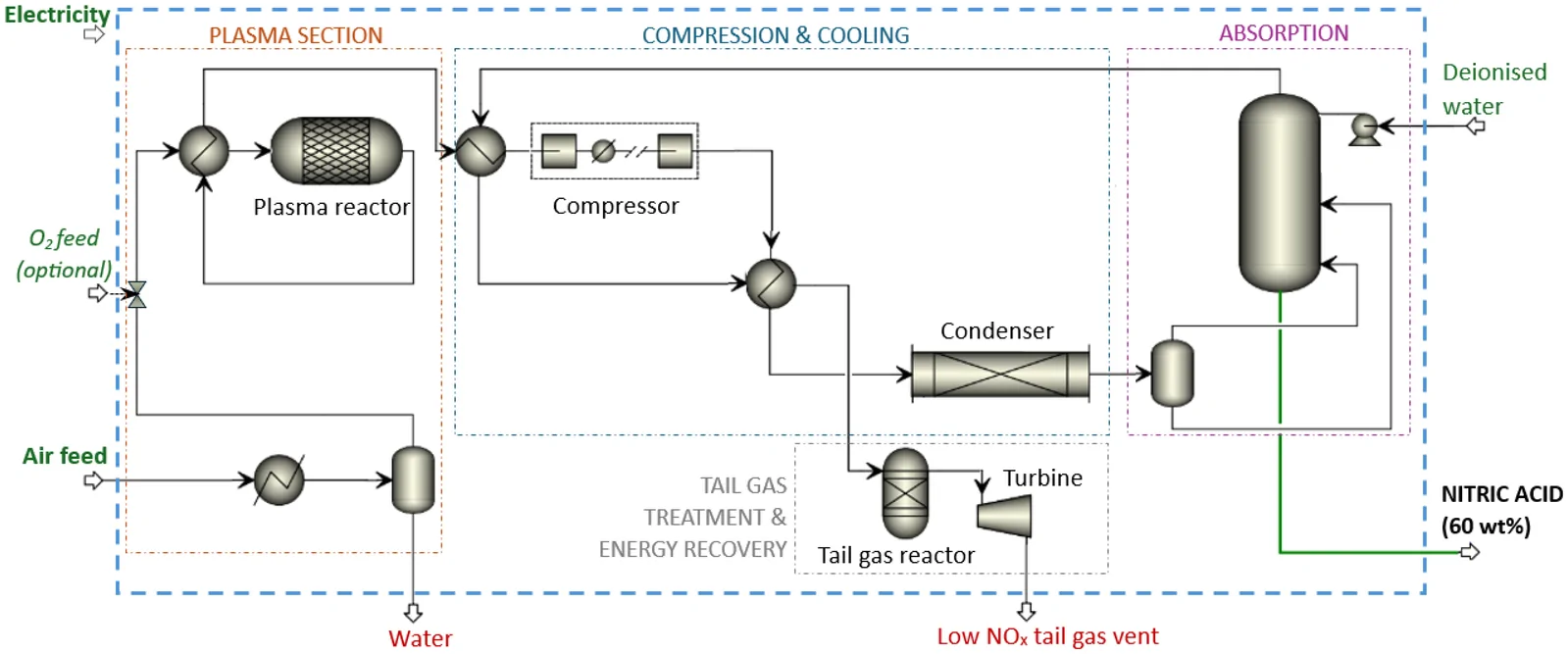
System boundaries of the plasma-based nitric acid production process.

The life cycle inventory (LCI) was developed using primary data from laboratory measurements for the plasma processes and system modelling in Aspen Plus 14 for the post-plasma sections (more details in the SI). Secondary data for upstream and background processes were obtained from the Ecoinvent database, adjusted using Australia-specific electricity data, and accounting for the monthly variation in the energy mix in 2025. The solar electricity profile was based on regional solar irradiance, inverter efficiency, and battery storage performance reported for the Roseworthy campus PV installation. Inventories for water, auxiliary chemicals, and heat transfer operations were modelled using Ecoinvent datasets.

In accordance with standard Ecoinvent methodology for chemical manufacturing processes, plant infrastructure (including plasma reactor bodies, electrodes, and power supply units) was modelled using the generic Chemical factory, organics infrastructure dataset as a proxy. This boundary choice reflects the current lack of empirical LCI data for plasma equipment. This maintains methodological parity with the conventional SMR–HB–Ostwald benchmark dataset, which similarly relies on generic Ecoinvent chemical plant proxies derived from aggregated European chemical site infrastructures rather than plant-specific hardware inventories.

Environmental impacts were assessed using ten impact categories selected to represent key environmental priorities in the chemical industry:^60–62^ global warming potential, terrestrial acidification, freshwater eutrophication, freshwater ecotoxicity, terrestrial ecotoxicity, photochemical ozone formation (human health), human carcinogenic toxicity, human non-carcinogenic toxicity, ozone depletion, and fossil resource scarcity. Special attention was given to categories most sensitive to energy and emission flows, such as global warming, photochemical ozone formation, and freshwater ecotoxicity, as these are strongly influenced by NO_*x*_ emissions, power generation, and water contamination.^62,63^ In addition, land use was included due to the relevance of photovoltaic electricity. For the characterisation of environmental impacts, the ReCiPe 2016 midpoint (H) method^64^ was selected due to its inclusion of the impact categories relevant to this study, along with its scientific robustness and global applicability.^65^

The results were benchmarked against the conventional SMR–HB–Ostwald process, using inventory data from the Ecoinvent database.^56,59^ For this study, the inventory was adapted to reflect the specific concentration modelled (60 wt%) and updated to include Australian utility data, ensuring regional relevance for water, electricity, heat, and grid-related impacts. The conventional route was modelled using the average Australian electricity mix, as major production plants, located in Kwinana and the Pilbara (Western Australia), Kooragang Island (New South Wales), and Yarwun, Moranbah, and Moura (Queensland),^66^ rely on fossil-heavy regional grids with renewable energy shares below 35%, closely mirroring the national average.^67^

A sensitivity analysis was performed to analyse how environmental impacts change due to the variance of the most relevant parameters, such as the EC plasma, oxygen generation, and plant capacity factors.

## 3 Results and discussion

### 3.1 Energy supply

The global horizontal irradiation at Roseworthy is approximately 1804 kWh per m^2^ per years, corresponding to an average of 4.9 kWh per m^2^ per day. As shown in Fig. 6, this resource exhibits marked seasonal variability, with monthly averages ranging from 2.15 kWh per m^2^ per day in June to 7.62 kWh per m^2^ per day in January. The clearness index, defined as the ratio of surface to extraterrestrial solar irradiation, indicates atmospheric clarity; values near 0.6 at Roseworthy indicate moderate and relatively stable solar availability throughout the year. This variability directly influences the availability of solar electricity throughout the year.

**Fig. 6:**
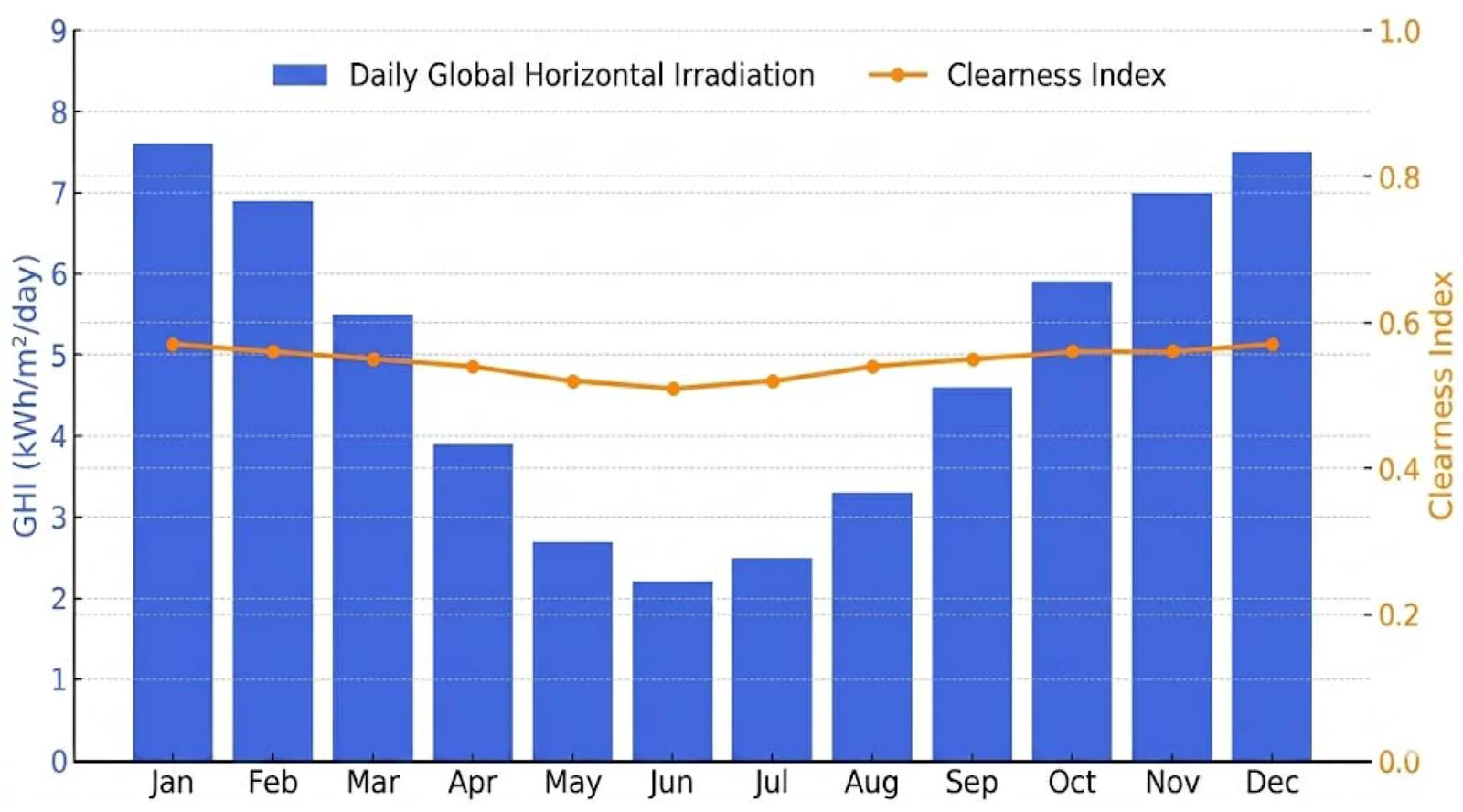
Monthly average global horizontal irradiation and clearness index at Roseworthy, Australia.

Measured PV generation data from the solar farm supplying the Roseworthy campus are presented in Fig. 7. The solid line represents the annual average daily profile based on half-hourly data, while the dashed lines correspond to January (maximum generation) and June (minimum generation).

**Fig. 7:**
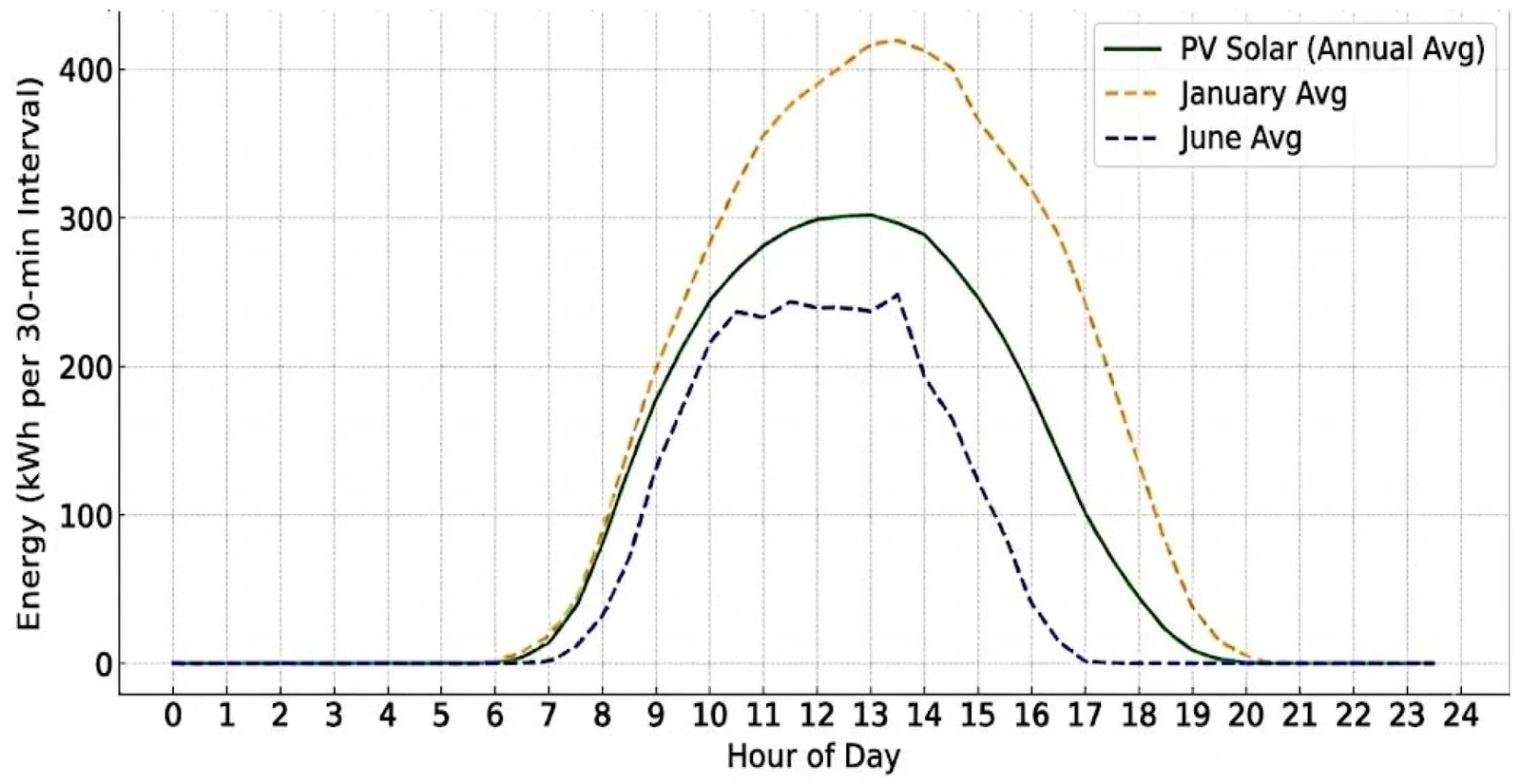
Photovoltaic electricity generation – hourly averages (annual, January, and June).

The PV generation profiles show pronounced seasonal and diurnal variability. In January, peak generation reaches approximately 420 kWh per 30 min interval (≈840 kW), whereas in June the peak decreases to 250–260 kWh per 30 min interval (≈500–520 kW), accompanied by a shorter generation window. The annual average profile lies between these extremes, with a peak slightly above 300 kWh per 30-minute interval. These differences highlight the strong dependence of available power on both season and time of day, which is a critical constraint for continuous process operation.

The profiles correspond to an annual generation of 1661 MWh, lower than the estimated potential of 2022 MWh per year due to operational curtailment and inverter clipping. This indicates that, despite sufficient solar resource, system-level constraints limit the effective utilisation of generated electricity. The current energy system is configured to maintain continuous base loads, particularly during night-time, primarily through grid imports to maintain minimum loads during the night of about 350 kW. Battery storage plays a complementary role, smoothing short-term fluctuations and extending the use of solar energy into early morning and evening periods, when grid electricity tariffs are typically higher.

Based on the generation and demand profiles, a plant capacity of approximately 500 kW (250 kWh per 30 min interval) can be supported under typical operating conditions. This capacity can be supplied for an average of 7.5 h per day directly from solar generation. Excess midday generation can be stored and later discharged to extend operation beyond peak solar hours. Under this dedicated setup, curtailment losses persist because peak solar generation exceeding the 500 kW plant demand remains unused. While sizing the plant closer to peak summer capacity (up to ∼700 kW) could absorb this excess energy and reduce curtailment, it would require higher capital expenditure and significantly increase reliance on grid electricity during low-solar periods.

Overall, the results indicate that while the available solar resource is sufficient to support a medium-scale plasma system, its utilisation is inherently constrained by temporal variability. This implies that plant operation must be partially flexible or supported by storage and grid integration, aspects further explored in the subsequent energy cost analysis. Based on the observed variability in solar generation, the implications for continuous plant operation were assessed by comparing the available PV supply with the projected plant demand.

### 3.2 Plant operation

To evaluate the operational feasibility of the proposed system, the available photovoltaic generation was compared with a constant plant demand of 500 kW over an annual operating time of 8000 h (∼91% capacity factor), reflecting realistic downtime for maintenance and operation. Table 2 presents the corresponding monthly allocation of solar- and grid-supported operating hours.

**Table 2:** Monthly operating hours supplied by solar and grid electricity for a 500 kW plant (8000 h per years)

Month	Plant operation (h per month)	Supplied by solar (h per month)	Supplied by grid (h per month)	Solar share (%)
Jan	679.5	434.0	245.5	63.9%
Feb	613.7	354.9	258.8	57.8%
Mar	679.5	313.2	366.2	46.1%
Apr	657.5	214.9	442.6	32.7%
May	679.5	153.8	525.7	22.6%
Jun	657.5	118.5	539.0	18.0%
Jul	679.5	142.4	537.1	21.0%
Aug	679.5	187.9	491.5	27.7%
Sep	657.5	253.5	404.0	38.6%
Oct	679.5	336.0	343.5	49.4%
Nov	657.5	385.8	271.8	58.7%
Dec	679.5	427.1	252.3	62.9%
**Total**	**8000.2**	**3322.0**	**4678.0**	**41.5%**

Assuming a plant demand of 500 kW and an annual operating time of 8000 h, the available PV generation of 1661 MWh per year would correspond to approximately 3322 equivalent full-load operating hours supplied by the Solar System, with the remaining 4678 h supplied by the grid. When distributed according to the monthly GHI profile, solar-supported operation is highest in summer months, reaching about 434 h per month in January and 427 h per month in December, while the minimum occurs in June at around 119 h per month. Under these assumptions, the Solar System would supply about 41.5% of the annual operating hours, with the grid covering the remaining 58.5%.

The results of the nitric acid process optimisation for the plant with both configurations are summarised in Table 3. The plasma air feed rate and post-plasma pressure were determined through a multi-objective optimisation targeting maximum HNO_3_ production and minimum specific energy cost under a total plant power constraint of 500 kW. While conventional nitric acid plants typically operate the cooling and absorption sections at 9–10 bar, lower operating pressures were found to be optimal in this case.

Energy costs in plasma-based HNO_3_ production plants at atmospheric pressure with 4 : 1 (air) and 1 : 1 feed ratios of N_2_ : O_2_

**Table:** 

Type of plasma reactor	GAP		RGA	
Feed ratio N_2_ : O_2_	4 : 1	1 : 1	4 : 1	1 : 1
NO_*x*_ concentration: vol% NO, vol% NO_2_	0.66, 0.54	0.69, 0.86	0.96, 0.37	1.12, 1.28
Plasma feed (L min^−1^)	14 400	7968	8849	4951
Post-plasma pressure (bar)	7.5	6.5	7.5	6.5
Production capacity (kg HNO_3_ h^−1^)	32.68	28.18	26.19	28.70

**Table:** 

Energy cost per section	(kW)	(kWh kg^−1^ HNO_3_)	(kW)	(kWh kg^−1^ HNO_3_)	(kW)	(kWh kg^−1^ HNO_3_)	(kW)	(kWh kg^−1^ HNO_3_)
Air feed	4.1	0.13	2.30	0.13	2.52	0.10	1.43	0.05
O_2_ feed	0	0	187.9	6.68	0	0	116.8	4.07
NO_*x*_ generation (plasma section)	434.0	13.28	275.7	9.80	456.7	17.44	360.4	12.56
Compression	84.1	2.57	43	1.53	51.8	1.98	26.6	0.927
Cooling	8.35	0.26	3.67	0.13	4.4	0.17	2.40	0.084
Absorption	0.008	0.0003	0.006	0.0002	0.007	0.0003	0.0063	0.0002
Electricity generation (turbine)	−30.3	−0.93	−12.5	−0.44	−15.6	−0.60	−7.5	−0.26
**Total**	**500**	**15.30**	**500**	**17.77**	**500**	**19.09**	**500**	**17.43**

Although increasing pressure enhances nitric acid production, it also increases compression energy requirements. Part of this additional energy is recovered through the expansion turbine, with higher pressures enabling greater electricity generation. However, despite this partial recovery, the net increase in compression-related energy outweighs the marginal gains in production beyond approximately 7.5 bar, leading to higher specific energy cost. Conversely, operating below 6 bar reduces compression demand but results in lower HNO_3_ output, which increases the specific energy cost due to the largely constant power requirement of the plasma reactor.

Therefore, operating pressures of 7.5 bar and 6.5 bar represent the optimal compromise between production rate and energy demand for the plasma 4 : 1 and 1 : 1 configurations, respectively, with the lower pressure in the latter driven by the higher NO_2_ concentration.

As shown in Table 3, in the air-fed configurations (4 : 1), the plasma reactor dominates the overall electricity demand for both technologies, while gas compression provides a secondary contribution that is partially offset by energy recovery in the expansion turbine. The RGA 4 : 1 configuration shows a higher total energy cost than the GAP 4 : 1 (19.09 *vs.* 15.30 kWh kg^−1^ HNO_3_), primarily due to its higher specific plasma energy demand (17.44 *vs.* 13.28 kWh kg^−1^ HNO_3_), despite achieving higher NO_*x*_ concentrations.

In the oxygen-enriched configurations (1 : 1), higher NO_*x*_ concentrations are achieved for both technologies, resulting in lower specific reactor energy demands. For the GAP 1 : 1 configuration, the additional energy required for O_2_ generation, which accounts for nearly 40% of total demand, outweighs the plasma efficiency gains, raising the total from 15.30 to 17.77 kWh kg^−1^ HNO_3_. In contrast, for the RGA 1 : 1 configuration, although O_2_ generation represents approximately 23% of total demand, the substantially reduced plasma energy consumption (from 17.44 to 12.56 kWh kg^−1^ HNO_3_) more than compensates for this additional cost, bringing the total down to 17.43 kWh kg^−1^ HNO_3_, slightly below the RGA 4 : 1 case. These differences in energy profiles across configurations and technologies are expected to play a key role in shaping the environmental performance of the system, as discussed in the next section.

### 3.3 Life cycle impacts of HNO_3_ production at atmospheric pressure

Fig. 8 presents the average contribution of the different inputs to the production of 1 kg of HNO_3_ over a typical year, assuming an electricity mix of 41.5% solar and 58.5% from the South Australian grid, and compares these results with those of the conventional SMR–HB–Ostwald process. To simplify the visualisation, the impacts associated with electricity generation in the expansion turbine are accounted for as a credit within the compression stage (post-plasma section), as this energy is recovered from the pressure increase during compression and offsets part of its electricity demand.

**Fig. 8:**
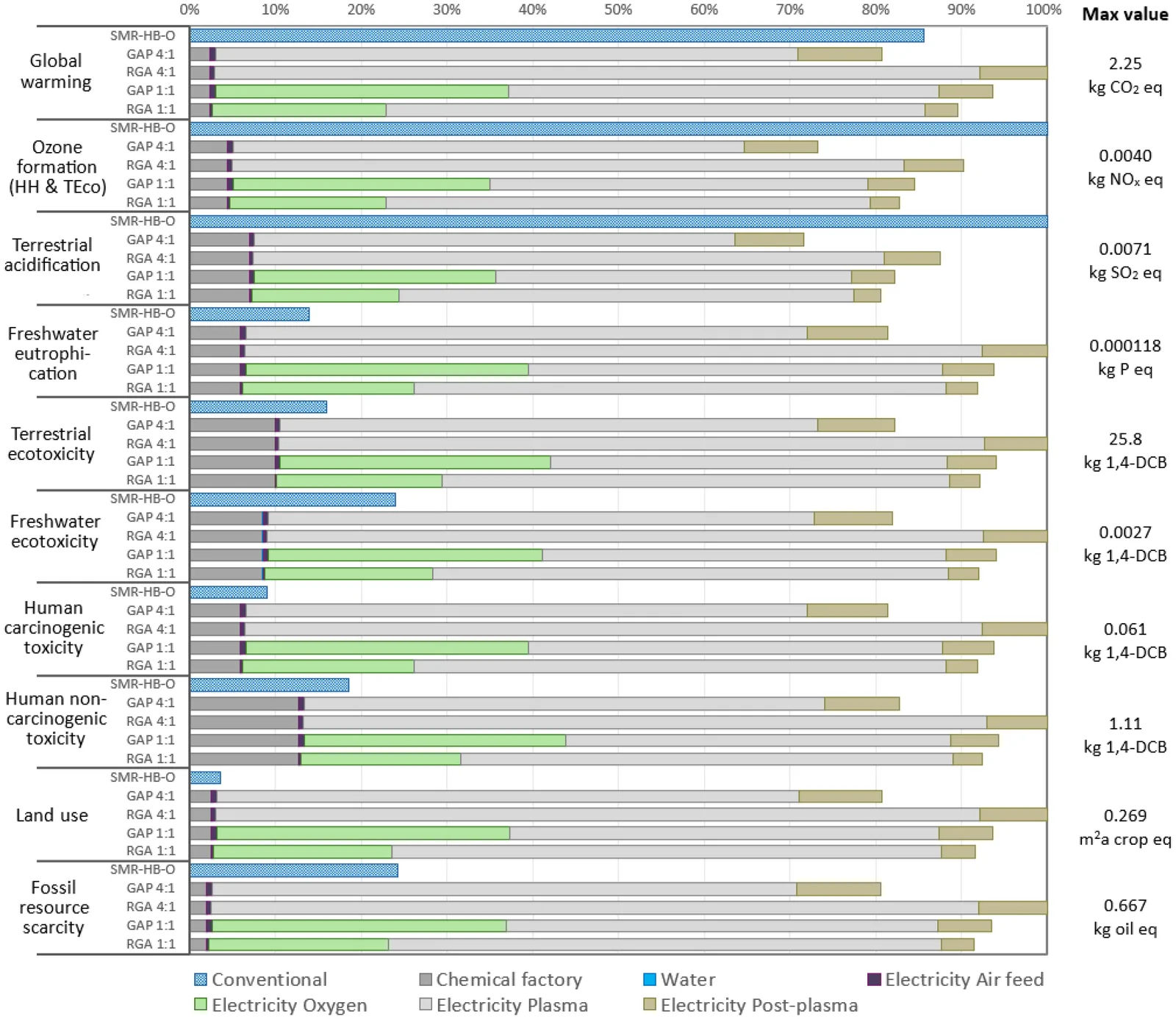
Life cycle impacts of the production of 1 kg of HNO_3_ (60 wt%) for the conventional process and plasma-based GAP and RGA routes at N_2_ : O_2_ ratios of 4 : 1 and 1 : 1 at atmospheric pressure. Each bar represents the impact as a percentage of the highest value for that category (normalised to 100%).

The plasma-based plants show lower impacts, with around 90% or less of the impacts shown by the conventional process for ozone formation (affecting both human health and terrestrial ecosystems) and terrestrial acidification. Within the global warming category, the GAP 4 : 1 configuration yields an emission factor of 1.82 kg CO_2_ eq. per kg HNO_3_, lower than the conventional route (with a factor of 1.93 kg CO_2_ eq. per kg HNO_3_). However, the other plasma configurations present higher emission factors of 2.11 kg CO_2_ eq. per kg HNO_3_ (GAP 1 : 1), 2.25 kg CO_2_ eq. per kg HNO_3_ (RGA 4 : 1) and 2.06 kg CO_2_ eq. per kg HNO_3_ (RGA 1 : 1).

These higher impacts in the conventional route in ozone formation and terrestrial acidification are mainly driven by the use of natural gas, which contributes not only to greenhouse gas emissions but also to NO_*x*_ and non-methane volatile organic compounds (NMVOCs), promoting ozone formation, and sulphur dioxide emissions, which lead to acid deposition and terrestrial acidification. In categories that might be affected by phosphate emissions, such as freshwater eutrophication, associated with the high coal- and lignite-based electricity generation in the average Australian electricity mix, the conventional SMR–HB–O route shows low impacts because this route is not as electricity-intensive as the plasma routes.

The plasma-based systems rely on a combination of on-site solar PV and the South Australian grid, which is virtually coal-free with a mix of wind (46.7%), solar (29.6%), natural gas (21.7%), and hydropower (1.9%). This lower carbon intensity of the electricity supply, together with the partial substitution by on-site renewable generation, contributes to the low impacts observed in climate- and air emission-related categories.

However, the electricity-intensive nature of the plasma-based plants leads to higher impacts in categories related to toxicity and land use. These impacts are primarily associated with the release of metals (*e.g.*, copper, silver) used in electrical infrastructure and photovoltaic systems, contributing to terrestrial ecotoxicity and human toxicity. It should be noted that these metal-related impacts capture only electrical and photovoltaic infrastructure; erosion-driven material loss from the plasma reactor electrodes and walls, observed qualitatively during operation but not yet quantified, is not included and would be expected to further increase this toxicity disadvantage. Increasing the share of solar PV does not substantially reduce these impacts, as photovoltaic systems themselves involve significant material-related emissions. In addition, land use impacts are considerably higher, by more than an order of magnitude, due to the area required for solar installations.

For these categories, reducing impacts is therefore more dependent on decreasing electricity demand (*e.g.*, improving plasma efficiency or increasing NO_*x*_ yields) than on increasing the share of renewable electricity. In contrast, fossil resource scarcity is influenced by both electricity demand and the composition of the supply. Despite partial substitution with renewable electricity, plasma-based systems still exhibit higher impacts due to their high energy requirements and the remaining fossil share in the grid. Given the influence of electricity supply on these results, the effect of improvements in plasma efficiency and temporal variations in the grid/solar mix is further examined.

A sensitivity analysis was conducted to assess the impacts resulting from lower EC plasma. Three progressive efficiency-improvement scenarios were considered, corresponding to 80%, 60%, and 40% of the current EC values listed in Table 1. In addition, a near-future target of 0.7 MJ mol^−1^ NO_*x*_ and a lower-bound value of 0.5 MJ mol^−1^ NO_*x*_ were considered based on reported gliding arc plasma performance limits.^17^ As these absolute targets represent different percentages of the current EC depending on the reactor and feed ratio, their relative values are also reported in Table 4.

**Table 4:** Energy cost in future scenarios for the atmospheric pressure plasma-based NO_*x*_ generation sections (EC plasma)

Type of reactor	N_2_ : O_2_ feed ratio	Current EC (MJ mol^−1^ NO_*x*_)	80% of current EC (MJ mol^−1^ NO_*x*_)	60% of current EC (MJ mol^−1^ NO_*x*_)	40% of current EC (MJ mol^−1^ NO_*x*_)	0.7 MJ mol^−1^ NO_*x*_ (% current EC)	0.5 MJ mol^−1^ NO_*x*_ (% current EC)
GAP	4 : 1	2.7	2.16	1.62	1.08	25.9%	18.5%
	1 : 1	2.4	1.92	1.44	0.96	29.2%	20.8%
RGA	4 : 1	4.2	3.36	2.52	1.68	16.7%	11.9%
	1 : 1	3.3	2.64	1.98	1.32	21.2%	15.2%

The impacts of these efficiency improvements on the global warming impact per kg of HNO_3_ produced are presented in Fig. 9. The results show a progressive reduction in carbon footprint with decreasing EC plasma. Reducing EC to 80%, 60%, and 40% of the current values decreases impacts by approximately 11–18%, 22–36%, and 32–54%, respectively. At 80% of the current EC, all four plasma configurations already achieve lower impacts than the SMR–HB–O benchmark. At the 0.7 MJ mol^−1^ NO_*x*_ target, impacts decrease to 0.579–1.307 kg CO_2_ eq per kg HNO_3_, corresponding to reductions of up to 74% relative to the respective base cases. The 0.5 MJ mol^−1^ NO_*x*_ scenario further reduces impacts to 0.482–1.212 kg CO_2_ eq per kg HNO_3_, highlighting the potential environmental benefits of future improvements in plasma energy efficiency.

**Fig. 9:**
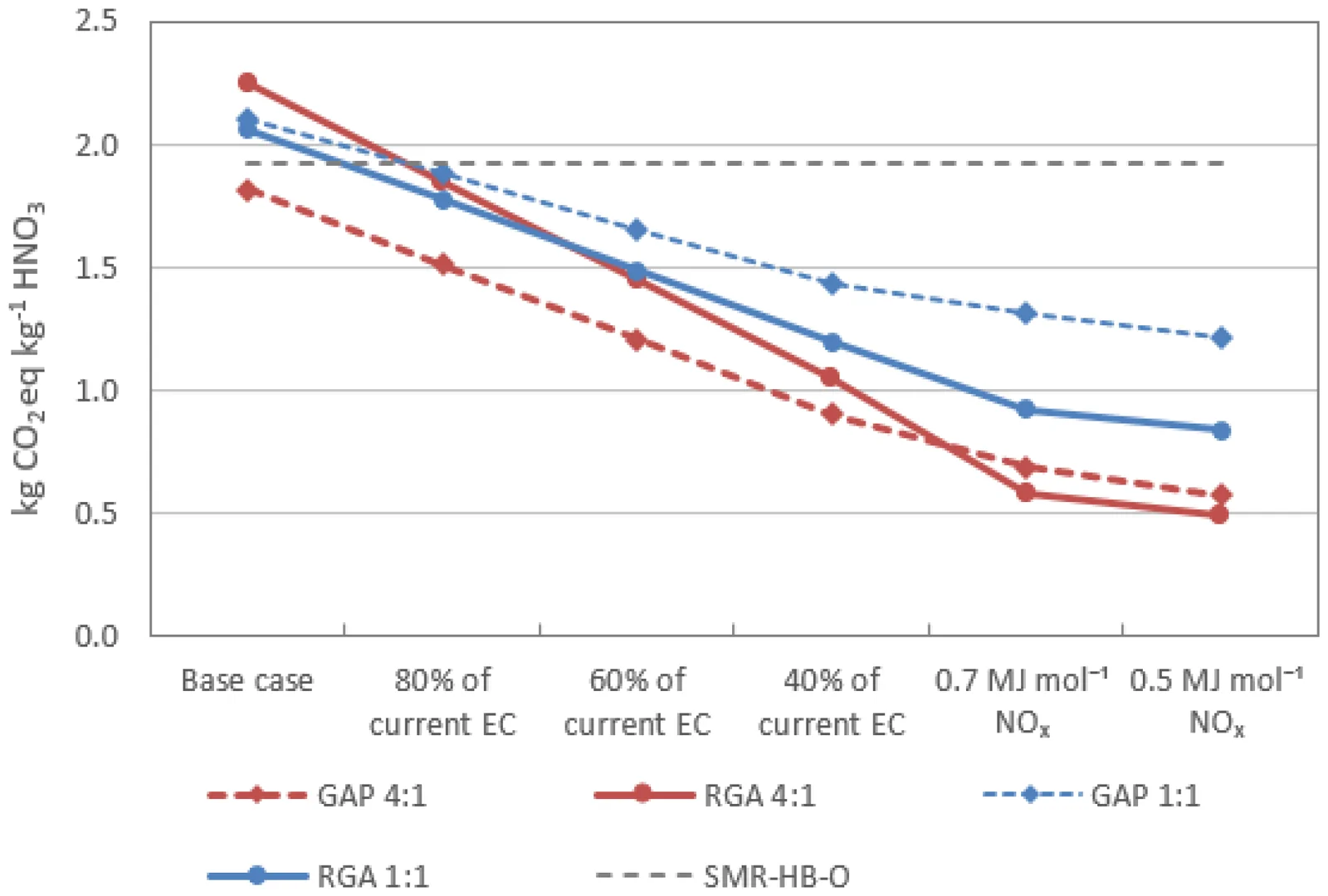
Global warming impacts as a function of the reduction in energy consumption for the NO_*x*_ generation section. Base case (41.5% solar share).

Nevertheless, without improvements in the EC plasma, it is still possible to reduce carbon emissions by increasing the solar share in the electricity supply, which can be seen during summer months. Fig. 10 presents the monthly results for the global warming impact category, enabling a more detailed assessment of this temporal variability.

**Fig. 10:**
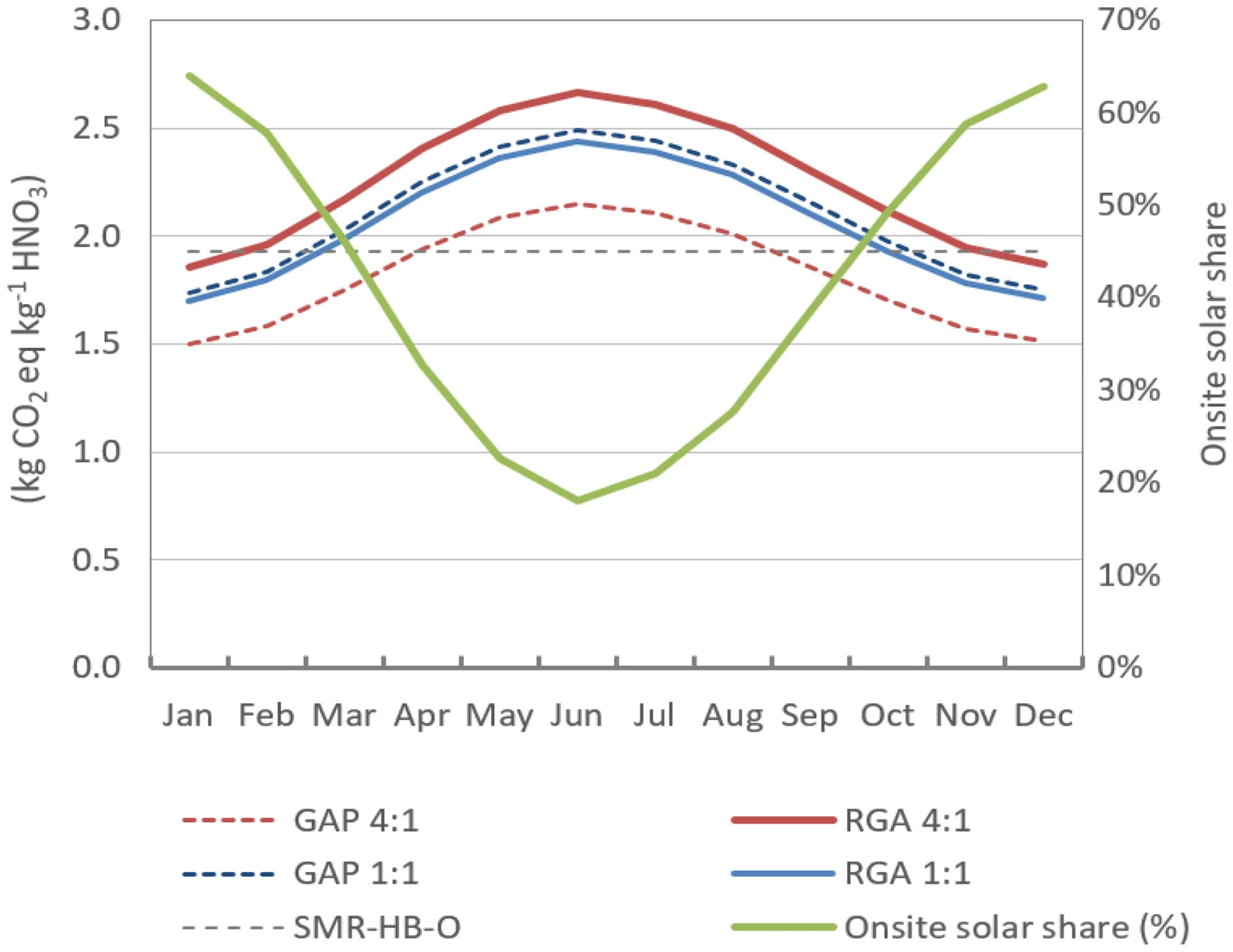
Global warming impacts of plasma-based nitric acid production (left axis) as a function of solar share (right axis) in different months.

An inverse relationship is observed between the solar contribution and the resulting global warming impacts. For the GAP 4 : 1 configuration, impacts range from a minimum of 1.50 kg CO_2_ eq. per kg HNO_3_ in January (64% solar share) to a maximum of 2.15 kg CO_2_ eq. per kg HNO_3_ in June (18% solar share). A similar trend is observed for the RGA 4 : 1 configuration, with values increasing from 1.85 kg CO_2_ eq. per kg HNO_3_ in January to 2.67 kg CO_2_ eq. per kg HNO_3_ in June. In both 4 : 1 configurations, this represents a seasonal variation of approximately 43%, highlighting the strong influence of the electricity mix on climate-related impacts.

For the 1 : 1 configurations, the same seasonal trend holds: GAP 1 : 1 ranges from 1.74 kg CO_2_ eq. per kg HNO_3_ in January to 2.49 kg CO_2_ eq. per kg HNO_3_ in June, while RGA 1 : 1 ranges from 1.70 to 2.44 kg CO_2_ eq. per kg HNO_3_ over the same period.

The monthly results reveal that the annual average global warming impacts reported in Fig. 8 (1.82–2.25 kg CO_2_ eq. per kg HNO_3_ across configurations, at 41.5% solar share) mask considerable seasonal variability. During months of high solar availability (austral summer, December–January), impacts fall approximately 17–18% below those annual averages, whereas during months of low solar availability (austral winter, June–July) they exceed them by approximately 18–19%, an asymmetry driven by the non-linear relationship between solar share and grid electricity carbon intensity.

These results confirm the sensitivity of plasma-based nitric acid production to the temporal variability of renewable electricity supply. This suggests that reducing reliance on grid electricity could improve environmental performance. One approach is to increase the share of on-site solar generation, which would require larger photovoltaic installations and significant investment in energy storage systems (*e.g.*, batteries), potentially leading to high capital costs. Alternatively, plant operation can be aligned with solar availability by reducing or halting production during periods of low renewable generation (*e.g.*, night-time). This strategy results in a lower plant capacity factor, the effects of which are evaluated in Fig. 11.

**Fig. 11:**
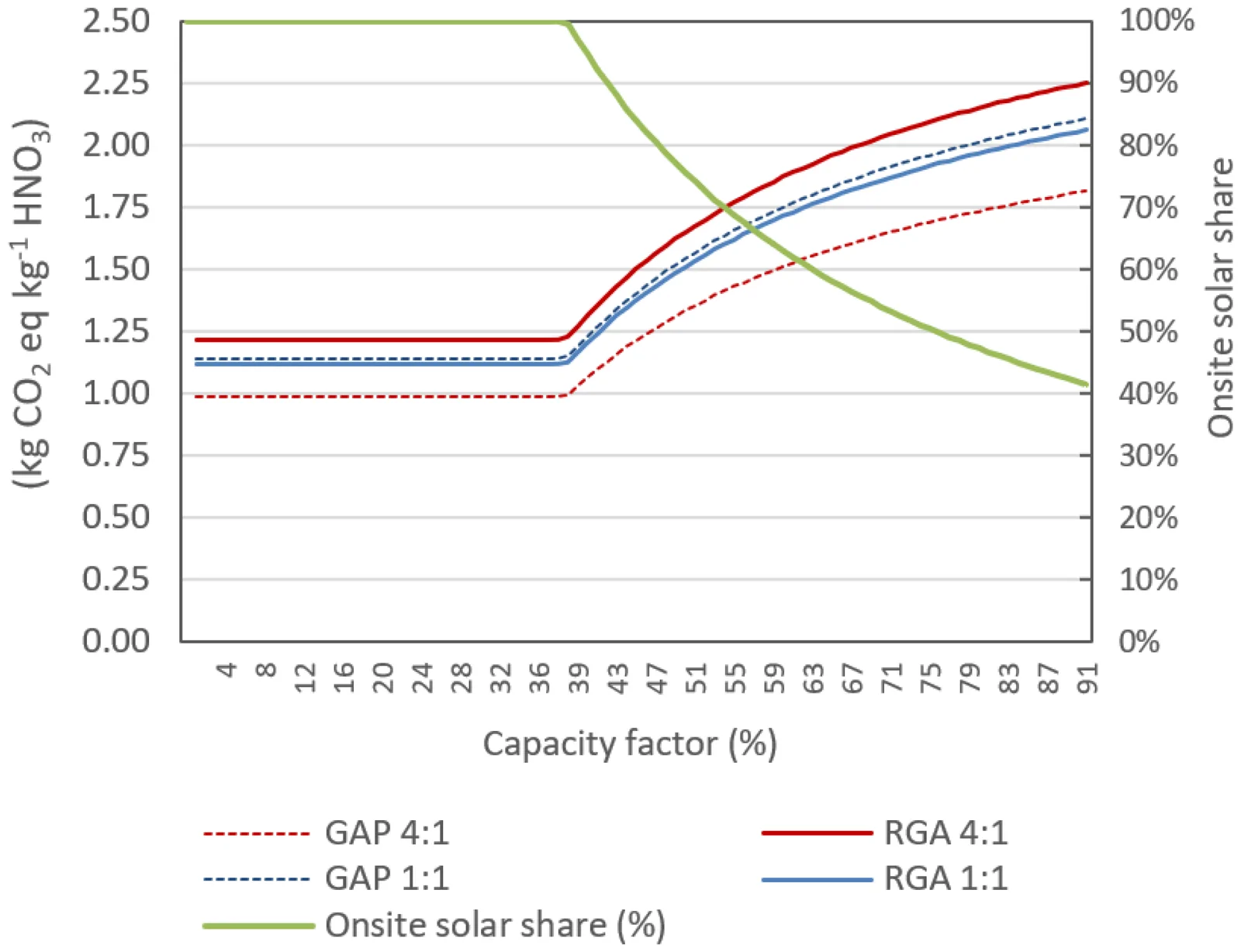
Global warming impacts (left axis) as a function of plant capacity factor and solar electricity share (right axis). Capacity factor represents the annual average with seasonally varying operation, based on Table 2 data.

A non-linear relationship is observed in Fig. 11, driven by the progressive incorporation of grid electricity as the capacity factor increases. It should be noted that the capacity factor considered here represents the annual average operating level, as plant operation is assumed to vary throughout the year, with higher utilisation during periods of high solar availability (*e.g.*, summer) and reduced operation during lower solar availability.

At plant capacity factors below 63% (*i.e.*, operating fewer than 5539 h per year), when the solar share is over 60%, all the plasma plant configurations emit lower carbon than the benchmark of 1.93 kg CO_2_ eq. per kg HNO_3._ At plant capacity factors below 37.8% (*i.e.*, operating fewer than 3323 h per year), the facility can run entirely on available solar energy, as indicated by the green curve reaching a 100% solar share in Fig. 11. Below this plant utilisation level, global warming impacts (expressed throughout in kg CO_2_ eq. per kg HNO_3_) become constant at their minimum values of approximately 0.99 for GAP 4 : 1, 1.14 for GAP 1 : 1, 1.22 for RGA 4 : 1, and 1.12 for RGA 1 : 1. However, low annual production may increase the unit production cost of HNO_3_, as capital investment and fixed operating costs are distributed over a smaller output, reducing cost-competitiveness.

Beyond the threshold of 37.8% capacity factor, grid electricity is introduced, leading to a steady increase in global warming impacts across all configurations. As the capacity factor increases further, the gap between the 4 : 1 and 1 : 1 configurations widens for both technologies, driven by the higher electricity demand of the oxygen-enriched system associated with oxygen production. As grid electricity is progressively introduced, its higher carbon intensity disproportionately affects the 1 : 1 cases, resulting in a steeper increase in global warming impacts compared to the 4 : 1 cases.

As evidenced in Table 3 and Fig. 8, the oxygen-enriched configuration exhibits higher environmental impacts, despite benefiting from reduced gas flow rates and lower compression requirements. This behaviour is largely driven by the additional energy demand for O_2_ production. The baseline energy demand for oxygen generation was estimated at 0.8 kWh kg^−1^ O_2_, based on pressure swing adsorption (PSA) with single-stage air compression. However, this value is subject to variability depending on the selected technology and plant scale. For instance, cryogenic air separation can achieve lower energy demands (∼0.2 kWh kg^−1^ O_2_), although it is typically limited to large-scale applications due to high capital costs.^68^ Conversely, higher energy requirements of up to 1.46 kWh kg^−1^ O_2_ have been reported for small-scale PSA systems.^69^

To account for this variability, a sensitivity analysis was performed considering an energy range between 0.2 and 1.4 kWh kg^−1^ O_2_, corresponding to ±0.6 kWh kg^−1^ O_2_ around the baseline value, presented in Fig. 12.

**Fig. 12:**
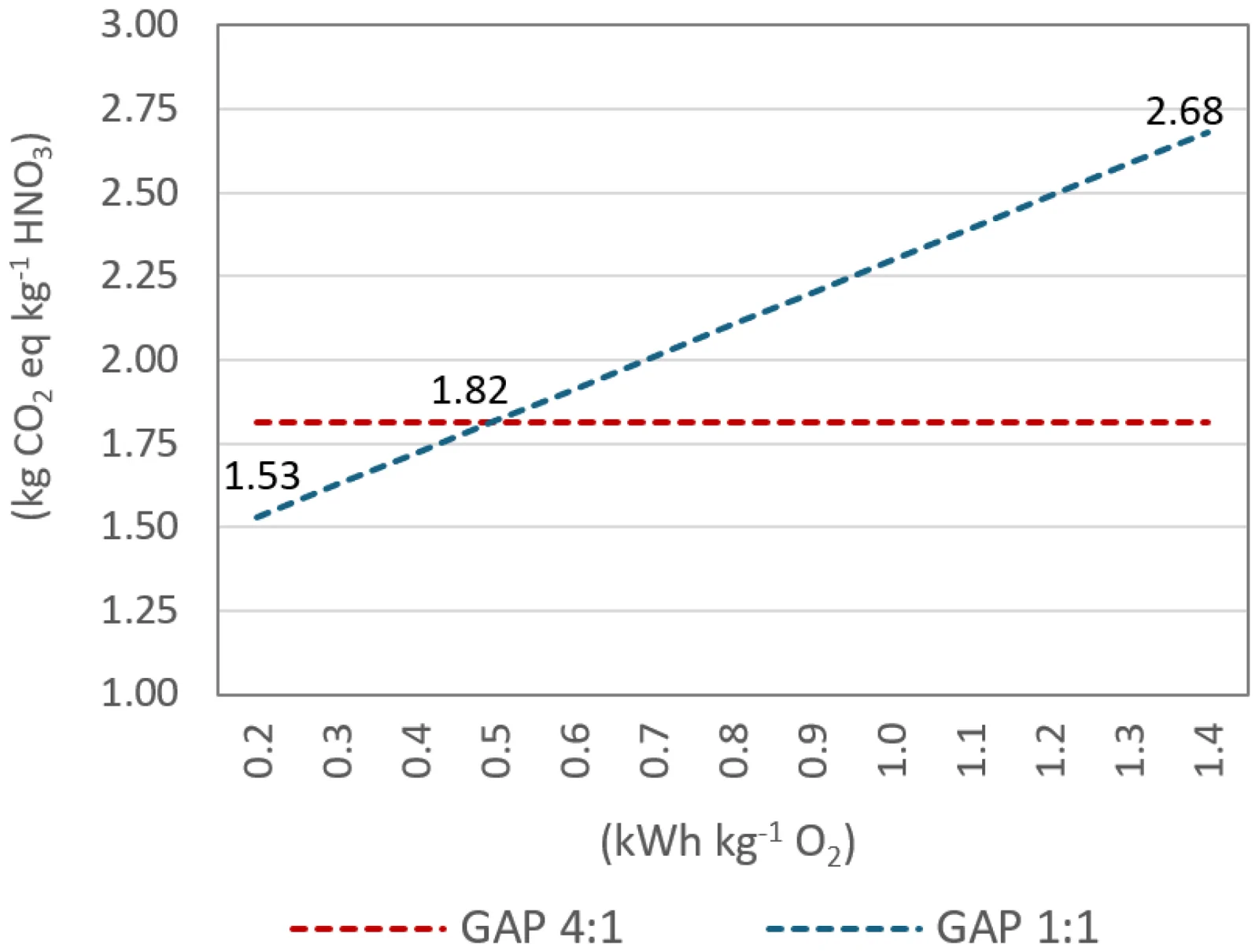
Sensitivity of global warming impacts to the energy demand for oxygen production in plasma-based nitric acid systems for the base case.

The oxygen-enriched configuration shows a strong sensitivity to the energy demand for oxygen production, with global warming impacts ranging from approximately 1.53 to 2.68 kg CO_2_ eq. per kg HNO_3_. From an operational perspective, reducing the specific energy demand for oxygen generation below the intersection point (≈0.5 kWh kg^−1^ O_2_) would enable the oxygen-enriched configuration to achieve lower impacts than the air-fed case. However, technologies capable of reaching such efficiencies may involve high capital costs, and their feasibility should therefore be evaluated through dedicated economic analysis. Beyond global warming, the performance of plasma-based routes in other environmental impact categories and potential strategies to address their limitations are examined in the following section.

These system-level variations highlight how regional grid characteristics shape environmental performance. More broadly, while the capacity factor and solar-share dynamics modelled here reflect the specific renewable supply constraints of South Australia, the global applicability of plasma-based nitric acid production will vary based on regional energy infrastructure. In regions with high grid decarbonisation or access to 100% green Power Purchase Agreements (PPAs), such as in parts of Europe, plant operation under near-zero carbon electricity is practical without relying on cost-prohibitive on-site battery storage.^70^ For regions without 100% renewable grid electricity, it is still possible to avoid fossil resources by integrating complementary renewable sources like wind energy and biomass, which can level diurnal supply fluctuations while offering lower infrastructure-related toxicity and land-use impacts than dedicated solar PV systems.^71^

### 3.4 HNO_3_ production at elevated pressures

Despite the potential benefits of oxygen-enriched gas feeds in reducing overall energy demand, the impacts of both plasma technologies would remain higher than those of the conventional pathway in the categories related to toxicity and land use, even under a hypothetical scenario of zero-cost oxygen input (*e.g.*, as a by-product from a secondary process). As evidenced in Fig. 8, removing the oxygen input contribution (light green bars) entirely would still leave the plasma-based impacts above the conventional ones in these categories. Further improvements must therefore target the efficiency of the plasma synthesis step itself (light grey bars).

One promising avenue is the operation of the plasma reactor at elevated pressures.^48^ Tsonev *et al.*, (2023)^41^ demonstrated that increasing the feed pressure from 1 to 4 bar raises NO_*x*_ concentrations from 1.33% to 2.40% (4 : 1) and from 2.40% to 4.90% (1 : 1), and, since plasma operating power remains virtually the same, it reduces energy cost from 4.2 to 2.3 MJ mol^−1^ NO_*x*_ and from 3.3 to 1.8 MJ mol^−1^ NO_*x*_ for the respective configurations.

Evaluating the true net benefit requires accounting for the additional feed compression energy, though this is partially offset by the reduced compression needed in the post-plasma section, given that the output is also delivered at elevated pressure. The detailed results for complete plants using RGA plasma at elevated pressure are presented in Table 5.

Energy costs in RGA-based HNO_3_ production at elevated pressure (4 bar) with 4 : 1 and 1 : 1 air feed ratios N_2_ : O_2_

**Table:** 

Feed ratio N_2_ : O_2_	4 : 1	1 : 1
NO_*x*_ concentration: vol% NO, vol% NO_2_	0.51, 1.89	0.29, 4.61
Plasma feed (L min^−1^)	9195	4648
Post-plasma pressure (bar)	6	6
Production capacity (kg HNO_3_ h^−1^)	50.52	54.77

**Table:** 

Energy cost per section		(kW)	(kWh kg^−1^ HNO_3)_	(kW)	(kWh kg^−1^ HNO_3_)
Feed	Air feed	2.55	0.050	1.33	0.024
	O_2_ feed			109.6	2.0
Plasma	Compression 1	30.0	0.59	15.2	0.28
	Cooling 1	0.83	0.016	0.43	0.008
	NO_*x*_ generation	466.4	9.22	372.2	6.79
Post-plasma	Compression 2	10.1	0.20	5.0	0.091
	Cooling 2	3.6	0.071	1.93	0.035
	Absorption	0.010	0.0002	0.010	0.0002
	Electricity generation	−13.5	−0.267	−5.5	−0.100
	**Total**	**500**	**9.88**	**500**	**9.13**

Compared to the atmospheric RGA-based plant reported in Table 3, which had plasma section energy costs of 17.44 and 12.56 kWh kg^−1^ HNO_3_ for the 4 : 1 and 1 : 1 configurations, respectively, elevated-pressure operation reduces the overall energy cost of the plasma sections (compression 1, cooling 1, and NO_*x*_ generation) to 9.83 and 7.15 kWh kg^−1^ HNO_3_, representing reductions of approximately 43% in both cases. Typically, higher pressure may increase the power consumption to sustain the plasma and thus reduce the gain in energy cost (as we have previously shown, *e.g.* for a feed gas flow rate of 4 L min^−1^)^41^ due to the increased energy losses (as heat) at long residence times. However, a higher flow rate (12 L min^−1^, used in this work) provides better thermal insulation of the plasma from the reactor walls and hence reduced heat losses. The power consumption consequently remains approximately independent of pressure.

Accounting for the full plant, the total energy cost drops from 17.44 to 9.88 kWh kg^−1^ HNO_3_ for the 4 : 1 configuration (a 43% reduction) and from 12.56 to 9.13 kWh kg^−1^ HNO_3_ for the 1 : 1 configuration (a 27% reduction), the latter being comparatively more modest due to the additional energy demand of the O_2_ feed compression. Additionally, the energy required in the post-plasma sections (compression 2, cooling 2, and absorption) to raise the gas from 4 to 6 bar is almost entirely offset by the energy recovered in the turbine. The resulting net energy demand of the post-plasma section is just 0.004 and 0.026 kWh kg^−1^ HNO_3_ for the 4 : 1 and 1 : 1 configurations, representing only 0.04% and 0.28% of the respective total energy costs of 9.88 and 9.13 kWh kg^−1^ HNO_3_. The environmental impact assessment for the elevated-pressure plants is presented in Fig. 13.

**Fig. 13:**
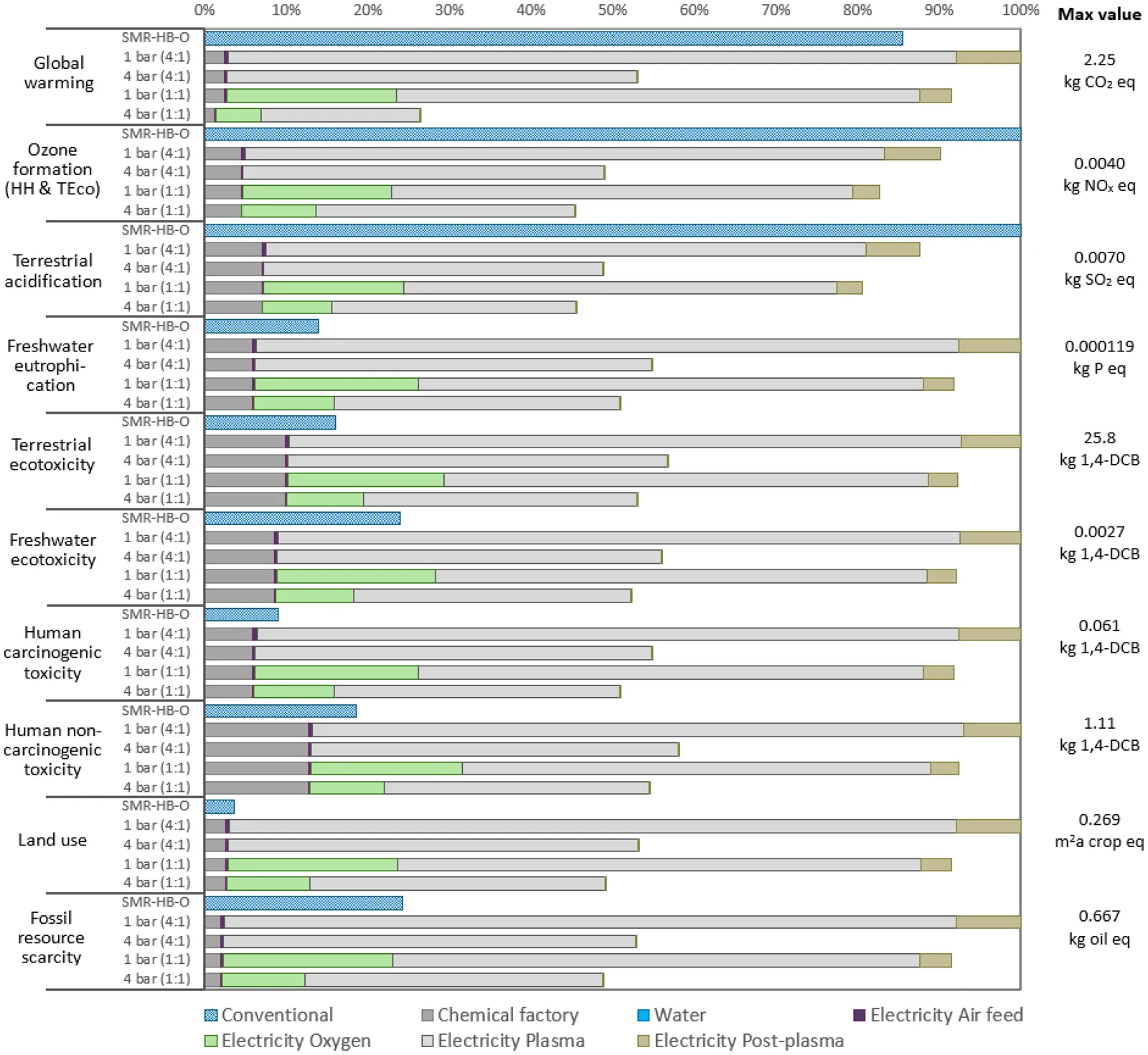
Life cycle impacts of the production of 1 kg of HNO_3_ (60 wt%) for the conventional process and RGA-based routes at N_2_ : O_2_ ratios of 4 : 1 and 1 : 1 at atmospheric pressure (1 bar) and at elevated pressure (4 bar). Each bar represents the impact as a percentage of the highest value for that category (normalised to 100%).

For the categories where plasma-based routes already outperformed the conventional process at atmospheric pressure (ozone formation and terrestrial acidification), elevated-pressure operation brings further substantial reductions, driven by the significantly lower electricity consumption of the plasma section and the near-elimination of the post-plasma energy burden (brownish bars), which becomes negligible at elevated pressure.

Within the global warming category, both RGA configurations at 4 bar obtain lower carbon emissions than the conventional route. The RGA 4 : 1 configuration at 4 bar achieves an absolute impact of 1.19 kg CO_2_ eq. per kg HNO_3_, compared to 2.25 kg CO_2_ eq. per kg HNO_3_ at atmospheric pressure, representing a 47% reduction. Similarly, the RGA 1 : 1 configuration drops from 2.06 to 1.10 kg CO_2_ eq. per kg HNO_3_, a reduction of 47%. These carbon factors represent drops of 38% and 43% relative to the conventional process. More broadly, the RGA 4 : 1 configuration at 4 bar reaches only 53%, 49%, and 49% of the maximum value for global warming, ozone formation, and terrestrial acidification, respectively. The 1 : 1 configuration follows the same trend, dropping to 26%, 46%, and 47%. For the rest of the impact categories, where the maximum impacts were obtained by the RGA 4 : 1 at atmospheric pressure, the 4 : 1 and 1 : 1 configurations at 4 bar presented approximately 55% and 50% of the impacts, respectively. As was observed at atmospheric pressure, the dominant contributors to these categories in the plasma configurations are the electricity inputs to the plasma section, whose reduced magnitude at elevated pressure is directly responsible for the improved performance.

Despite these substantial reductions, the elevated-pressure plasma configurations still exceed the conventional SMR-HB-O process in toxicity-related categories, land use, and fossil resource scarcity. Land use in particular remains significantly higher, by more than an order of magnitude for the 4 : 1 configuration, due to the continued reliance on solar PV electricity.

Overall, elevated-pressure operation represents a significant step forward for the environmental performance of plasma-based nitric acid production across all impact categories. The reduction in plasma energy cost propagates consistently through both energy-related and material-related impact categories, narrowing the gap with the conventional process substantially. Nevertheless, in the toxicity and land use categories, the conventional process retains a clear advantage, indicating that further improvements in plasma efficiency remain necessary for plasma-based routes to become fully competitive across all environmental dimensions.

These results show that both atmospheric and elevated pressure plasma configurations reduce most environmental impacts per unit of HNO_3_ compared to the conventional route. However, gliding arc plasma has a lower technology readiness level (TRL) than the electrified HB pathway, which combines green ammonia from water electrolysis and HB loop with a renewably powered Ostwald process. Unlike plasma, green ammonia is already validated at pilot and commercial scales. The Green Ammonia Working Group^72^ reports an average electricity consumption of 10.6 MWh per t NH_3_, distributed among H_2_ production *via* water electrolysis (91.5%), HB synthesis (6.1%), nitrogen separation (2.3%), and water purification (0.01%).

Producing one tonne of HNO_3_ (60 wt%) at a standard 96% conversion efficiency requires 175.2 kg of green NH_3_. For highly optimised, large-scale systems, this equates to 1.86 kWh kg^−1^ HNO_3_ upstream and ∼0.06 kWh kg^−1^ HNO_3_ for the Ostwald and abatement block,^73^ totalling a benchmark of ∼1.9 kWh kg^−1^ HNO_3_. This is substantially lower than the lowest plasma-route benchmark achieved in this study (9.13 kWh kg^−1^ HNO_3_). However, as mentioned above, plasma technology is still at a lower TRL, and there is still substantial room for improvement. This can be done by improving reactor design (which is now possible due to the development of predictive plasma reactor computational fluid dynamics models for NO_*x*_ production from air) as well as by post-plasma quenching (fast cooling of the plasma effluent to avoid back-reactions) and heat recovery.^28^

Furthermore, when matching the decentralised, small-scale nature of plasma systems, the electrified HB-Ostwald route suffers severe energy penalties. Downscaling compromises compressor efficiencies and restricts heat recovery. Anastasopoulou *et al.* (2020)^74^ reported that a standalone small-scale renewable H_2_-based ammonia plant requires 12.8 MWh per t NH_3_, which includes 7.35 MWh for electrolysis, 0.514 kWh for air separation, and 4.89 MWh for the HB synthesis loop. Additionally, the process demands 17.7 MJ of thermal cooling energy per tonne of NH_3_, which adds approximately 1.64 MWh per t NH_3_ using an industrial electric chiller network.

This brings the true cumulative electricity demand of small-scale green NH_3_ to 14.4 MWh per t NH_3_. When allocated to downstream nitric acid production alongside the downscaled Ostwald block penalty (0.12–0.18 kWh kg^−1^ HNO_3_),^73^ the total electricity requirement for the small-scale electrified HB-Ostwald route reaches approximately 2.64–2.70 kWh kg^−1^ HNO_3_. While these small-scale inefficiencies widen the energy gap compared to large-scale baselines, this thermochemical route still represents a lower energy barrier than the plasma pathways.

Nevertheless, the commercial competitiveness of this downscaled HB route is also limited by high electrolyser capital costs, expensive catalysts, and operational rigidity. The HB loop requires continuous operation and takes hours or days to reach a steady state,^75,76^ necessitating capital-intensive NH_3_ storage to buffer intermittent renewable power or changing demand. Conversely, atmospheric-pressure plasma routes offer rapid start-stop capabilities, bypassing these storage and operational bottlenecks entirely.^77^ Moreover, future improvements in energy efficiency are more expected for plasma technology than for electrified HB synthesis. If the lower-bound value of 0.5 MJ mol^−1^ NO_*x*_ detailed in Table 4 is considered, the total energy cost for the atmospheric pressure technologies detailed in Table 3 would drop as low as 3.73 kWh kg^−1^ HNO_3_ and for the elevated pressure technologies detailed in Table 5 would drop as low as 2.66 kWh kg^−1^ HNO_3_, matching the energy cost of the small-scale electrified HB-Ostwald route.

It is also worth noting that the SMR–HB–Ostwald benchmark, like the electrified HB-Ostwald route above, reflects factory-gate emissions and excludes transport to the point of use. As nitric acid plants in Australia are located in other states (Western Australia, New South Wales, and Queensland, as noted above), conventional HNO_3_ would need to be transported long distances to reach South Australia, unlike the decentralised, on-site plasma-based systems evaluated here. To our knowledge, no published life cycle data exist for nitric acid transport, so we use a proxy from our previous study on ammonia transport emissions in Australia,^78^ which reported 0.33–0.87 kg CO_2_ eq. per kg NH_3_ depending on transport mode. NH_3_ requires refrigerated/pressurised transport, adding energy overhead not applicable to HNO_3_ (a liquid at ambient conditions), so this figure is likely an upper-bound proxy; both are nonetheless hazardous liquids transported by tanker, with emissions dominated by vehicle fuel consumption. Adding this range to the factory-gate benchmark (1.93 kg CO_2_ eq. per kg HNO_3_) gives a transport-inclusive estimate of 2.26–2.80 kg CO_2_ eq. per kg HNO_3_—higher than all plasma configurations under base-case assumptions. This suggests the case for decentralised plasma-based HNO_3_ production may be even stronger, and conservatively so, than the factory-gate comparison indicates.

Beyond the environmental and energy considerations discussed above, the practical implementation of plasma-based nitric acid production at industrial scale raises legitimate questions of engineering feasibility. If the full-scale GAP 4 : 1 plant (requiring a total feed rate of ∼14 400 L min^−1^) were built from unmodified lab-scale reactor units, each operating at only 241 W (10 L min^−1^ feed; Table 1), this would imply numbering-up hundreds of units. Clearly, a design with only a few upsized units would be more practical. Power supply units and single-reactor arc plasma systems in the 100 kW to several-MW range have already been demonstrated for various arc-plasma processes at industrial scale, including nitric acid production by the Birkeland–Eyde process.^20,79–81^ Rotating-arc torch designs, directly analogous in operating principle to the RGA reactor used in this study, were scaled from 150 kW to 3 MW per unit for methane pyrolysis, with arc rotation improving stability and heat distribution while reducing electrode wear.^80^ Similarly, the Birkeland–Eyde plasma reactor for nitric acid production was based on applying a magnetic field at right angles to deflect the plasma arc, so that it could spread through the reactor, allowing much larger reactor size without substantial gas bypass.^36^ On this basis, a more realistic scale-up pathway for the present system is to upsize the individual GAP/RGA reactor toward the order of ∼100 kW, well within the range already demonstrated for various arc-plasma applications, including plasma-based nitric acid production, which would allow the plasma power of the full-scale GAP 4 : 1 plant to be met using on the order of only five parallel reactor modules. This is consistent with the scale-out strategy already being pursued at pilot scale for GAP technology specifically by the D-CRBN spin-off company of UAntwerp/PLASMANT, which currently operates multiple low-power (∼1 kW) GAP modules in parallel for CO_2_ conversion and has indicated plans to increase individual module power as part of its ongoing development.^52^

This pathway is contingent on the specific energy consumption of the GAP/RGA reactor remaining approximately constant as the individual unit is upsized, an open engineering question specific to gliding/rotating arc geometry (electrode gap, gas velocity required for arc stability, heat management), which requires further investigation. A dedicated techno-economic and engineering feasibility study, including reactor and electrode design at the 10–100 kW scale, is identified as a priority for future work.

## 4 Conclusions

This study evaluates the environmental performance of plasma-based nitric acid production for two different GA reactors, *i.e.*, GAP and RGA, under air-fed (4 : 1) and oxygen-enriched (1 : 1) configurations, benchmarking against the conventional SMR-HB-Ostwald process. The results demonstrate that plasma-based systems can substantially reduce impacts in key categories such as global warming, ozone formation, and terrestrial acidification, primarily due to the avoidance of fossil-based ammonia synthesis. However, their environmental performance is strongly influenced by electricity demand and the source, composition, and temporal variability of the electricity supply, with the plasma reactor representing the dominant energy consumer and, consequently, the main contributor to impacts.

The analysis highlights the critical role of electricity sourcing and operational strategy. Temporal variability in solar availability leads to significant seasonal fluctuations in environmental performance, with variations of up to approximately 43% in global warming impacts over the year. While increasing the share of renewable electricity can improve environmental performance, it requires either expanded photovoltaic capacity and energy storage or adjustments in plant operation. In this context, reducing the plant capacity factor to align production with solar availability enables operation under fully renewable conditions with minimum impacts, but introduces trade-offs related to reduced production output and potential economic implications, which are beyond the scope of this study.

The oxygen-enriched configuration offers process advantages through higher NO_*x*_ yields and reduced gas throughput, but these benefits are offset by the additional energy required to generate oxygen. Its environmental performance is highly sensitive to oxygen production efficiency, with a threshold of approximately 0.5 kWh kg^−1^ O_2_ below which it becomes environmentally favourable compared to the air-fed configuration. However, achieving such low energy demands for oxygen production may require advanced or capital-intensive technologies, and their feasibility should therefore be evaluated through dedicated techno-economic analysis. Regardless of oxygen sourcing strategy, the plasma energy demand remains the primary driver of impacts, particularly in toxicity and land use categories, where plasma-based routes exceed the conventional process even under a hypothetical scenario of zero-cost oxygen input.

Importantly, operating the RGA plasma reactor at elevated pressure (4 bar) represents a promising pathway to address this limitation. Pressure intensification increases NO_*x*_ concentrations and reduces energy consumption per mole of NO_*x*_ produced, resulting in total plant energy reductions of 43% and 27% for the 4 : 1 and 1 : 1 configurations, respectively, relative to atmospheric operation. These energy savings propagate across all impact categories, bringing global warming impacts down to 1.19 and 1.10 kg CO_2_ eq. per kg HNO_3_ for the 4 : 1 and 1 : 1 configurations, respectively, reductions of 38% and 43% relative to the conventional process. In the toxicity and land use categories, elevated-pressure operation delivers substantial improvements, roughly halving the normalised impacts compared to atmospheric pressure, though the conventional process retains an advantage in these categories.

Taken together, these findings indicate that plasma-based nitric acid production has clear potential as a low-carbon alternative to conventional routes, provided that improvements in plasma efficiency are achieved. Elevated-pressure operation emerges as a particularly effective lever, simultaneously reducing energy consumption, global warming impacts, and material-related environmental burdens. Nevertheless, full environmental competitiveness across all impact dimensions will require further advances in plasma reactor performance, optimisation of oxygen generation where applicable, and integration with low-carbon electricity systems. Future work should incorporate techno-economic assessment and explore system-level optimisation strategies to support the scalable deployment of this technology.

## Author contributions

J. Osorio-Tejada: writing – original draft, visualisation, investigation, conceptualisation, methodology. Y. Gorbanev: visualisation, investigation, writing. V. Kenjle: visualisation, data curation. A. Bogaerts: writing – reviewing and editing. V. Hessel: writing – reviewing and editing.

## Conflicts of interest

There are no conflicts to declare.

## Supplementary Material

GC-028-D6GC04056J-s001

## Data Availability

The data supporting this article have been included as part of the supplementary information (SI). Supplementary information: detailed process modelling. See DOI: https://doi.org/10.1039/d6gc04056j.
