# Supplementary material for: Environmental analysis of plasma-based nitrogen fixation powered by renewable energy
Source: Green Chem. 2026 Oct 20;28(42):16873–90. doi: 10.1039/d6gc04056j (PMC13681104; doi:10.1039/d6gc04056j)
Supplement: Supplementary file 1 [file GC-028-D6GC04056J-s001.pdf]

Supplementary Information (SI) for Green Chemistry.
This journal is © The Royal Society of Chemistry

Electronic Supplementary Information file for the article:

Environmental Analysis of Plasma-Based Nitrogen Fixation Powered by Renewable Energy

Jose Osorio-Tejada,*[a] Yury Gorbanev,[b c] Vikram Kenjle,[d] Annemie Bogaerts,*[b c] Volker Hessel [a d]

[a.] *School of Engineering, University of Warwick, United Kingdom.*
[b.] *Research group PLASMANT and PLASMA Center of Excellence, Department of Chemistry, University of Antwerp, Belgium.*
[c.] *Electrification Institute, University of Antwerp, Belgium.*
[d.] *School of Chemical Engineering, Adelaide University, Australia*

* *Corresponding authors: jose.osorio-tejada@warwick.ac.uk and annemie.bogaerts@uantwerpen.be*

This Electronic Supplementary Information (ESI) describes the detailed process modelling for the atmospheric and elevated pressure plasma-based plants

Plasma-based process: Description and model development

The model was implemented in Aspen Plus® V14 using the ELECNRTL property method for electrolyte systems and the NRTL-RK method for gas-phase simulations. The overall process consists of four main sections, as depicted in Figure 5 of the main text. The detailed equipment and process flows for each plant configuration are presented in Figure S1 and Figure S2.

(i) **Plasma section**: NO and NO_2 are generated in a plasma reactor, simulated as a stoichiometric reactor (Rstoic) with fractional conversion.

- Primary reaction: N_2+O_2⇌$2NO$
- Secondary reaction: $2NO+O_2$⇌$2NO_2$
- Operating conditions: 100°C, 1.01 bar.

(ii) **Compression and Cooling:** The plasma outlet is compressed to 6.0 – 7.5 bar and cooled using a heat exchanger (Heat-EX2) integrated with the tail gas stream to improve energy efficiency. Further cooling is achieved via a condenser block, which reduces the gas temperature before it enters the nitric acid absorber. This block is simulated in a Plug flow reactor (RPlug).

- Kinetic-driven reactions:
  - $2NO+O_2$⇌$2NO_2$ (NO oxidation)
  - $2NO_2$⇌N_2O_4 (NO_2 dimerization)
  - $3NO_2+H_2O$→$2HNO_3+NO$ (small acid formation)

- Reaction rate multipliers:
  - NO oxidation: 1
  - NO_2 dimerisation: 10
  - Acid formation: 8,000
- Heat transfer: UA = 1,934 J/s·K.

Although the underlying gas-phase NO/NO₂ chemistry is reversible, in Aspen these reactions were implemented as forward kinetic steps with user-defined rate constants. Only the NO₂ ⇌ N₂O₄ dimerisation was treated explicitly as an equilibrium reaction.

(iii) **Absorption:** The absorber column is implemented as a RadFrac block with 49 equilibrium stages and a partial condenser. The unit facilitates the absorption of NO₂ in water to produce nitric acid via the reactions:

- 3 NO₂ + H_2O → 2 HNO_3 + NO
- NO + ½ O_2 ⇌NO_2 (regeneration loop)

Water is fed at a controlled rate (calculated based on HNO₃ concentration targets, up to 60 wt%), and condenser conditions were set to maintain the outlet at ~30 °C. The condenser duty was set via a utility exchanger (using STEAMNBS data), and the water stream flow was tuned to maintain the target acid concentration: S-7, NOₓ-rich gas feed (stage 49 - bottom); S-8, water condensate (stage 44 - middle); and S-9B, fresh water makeup (stage 1 - top) for dilution control of final acid concentration and bleaching of residual NOₓ via enhanced gas-liquid contact.

The bleaching stage was integrated directly into the Radfrac absorption column model through a dedicated pump-around loop. This configuration enhances the removal of residual NO and NO_2 from the nitric acid product by recirculating cooled acid to the lower stages of the column. The objective of this internal bleaching setup is to improve product purity and stability by promoting additional gas–liquid contact and facilitating further absorption of dissolved nitrogen oxides. In Aspen Plus, this functionality is achieved without requiring a separate bleaching column. This modelling approach is consistent with configurations in industrial nitric acid plants, where internal circulation and recirculated acid loops are used to achieve high absorption efficiency and improve product acid quality.[1,2]

(iv) **Tail Gas Treatment and Energy recovery:** The tail gas from the absorber (S-11A, 34 °C, 6.15 bar) is routed to the RGibbs thermal reactor, which receives the stream as S-11D at 91 °C.

---

[1] G. R. O. Nascimento and J. V. H. Dangelo, *Chem. Eng. Process. - Process Intensif.*, 2024, **196**, 109662.

[2] I. B. Chatterjee and J. B. Joshi, *Chem. Eng. J.*, 2008, **138**, 556–577.

Post-reaction gas (S-12A) is sent through a turbine to recover mechanical power before being vented as stream S-12C. The total NOx levels are reduced to about 3E-07 mole fraction, consistent with regulatory standards. In Australia, emission limits for NOx (as NO_2 equivalent) from nitric acid plant tail gas vary by state and are typically specified in Environmental Protection Licences (EPLs), such as the limit set for the Yara nitric acid production plant of 103 mg/m^3 (≈ 50 ppmV) in Western Australia.[3] After tail gas treatment, the vent gas in the modelled plant has an NOx concentration below 1 ppmV, meeting the strictest environmental regulations.

[3] YPN, *Air Quality Management Plan Technical Ammonium Nitrate Plant. 650-200-PLN-YPN-0002 Rev D*, Karratha, 2022.

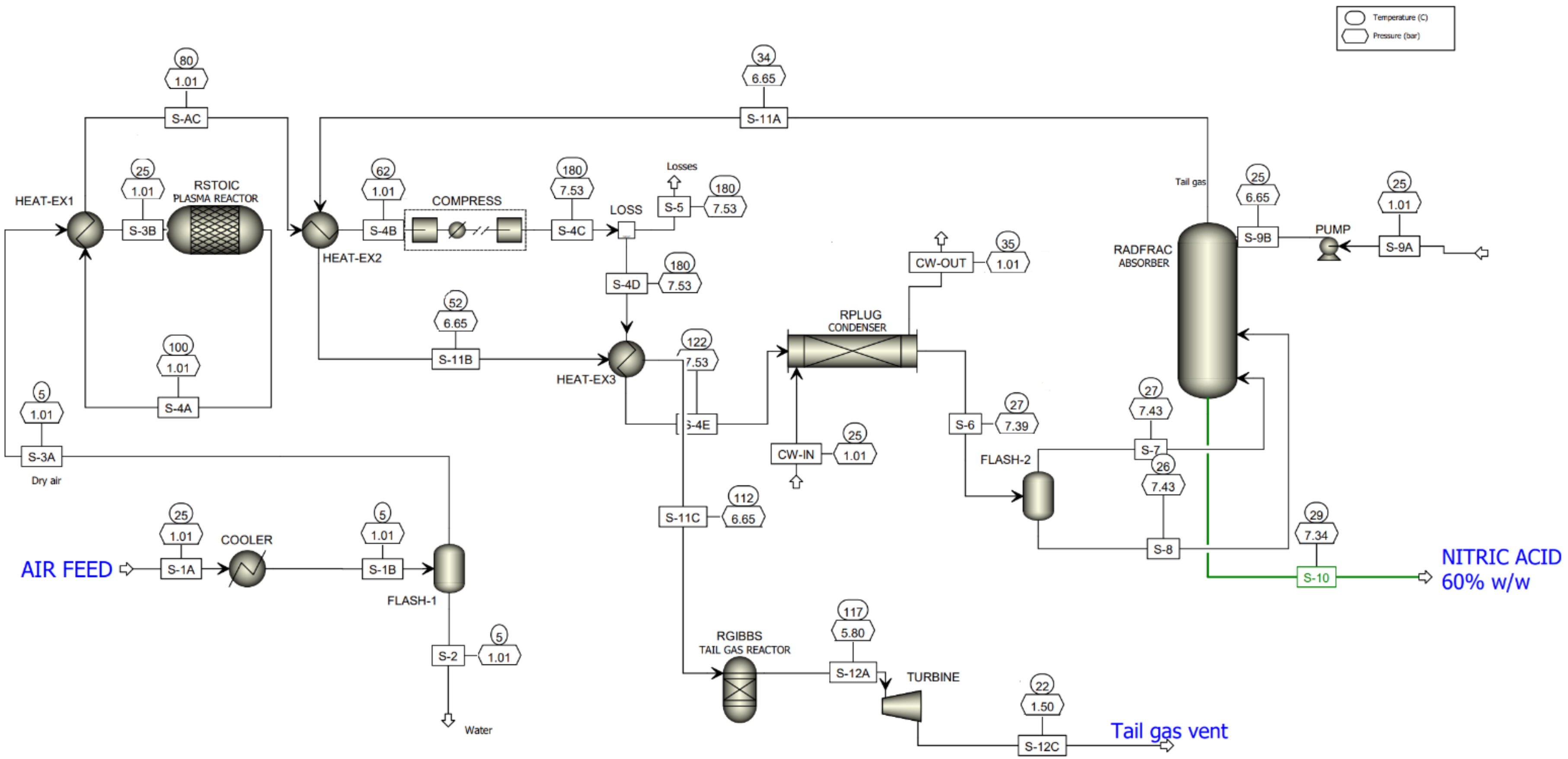

Figure S1. Nitric acid production plant using an atmospheric glow discharge plasma reactor with 4:1 N_2:O_2 ratio

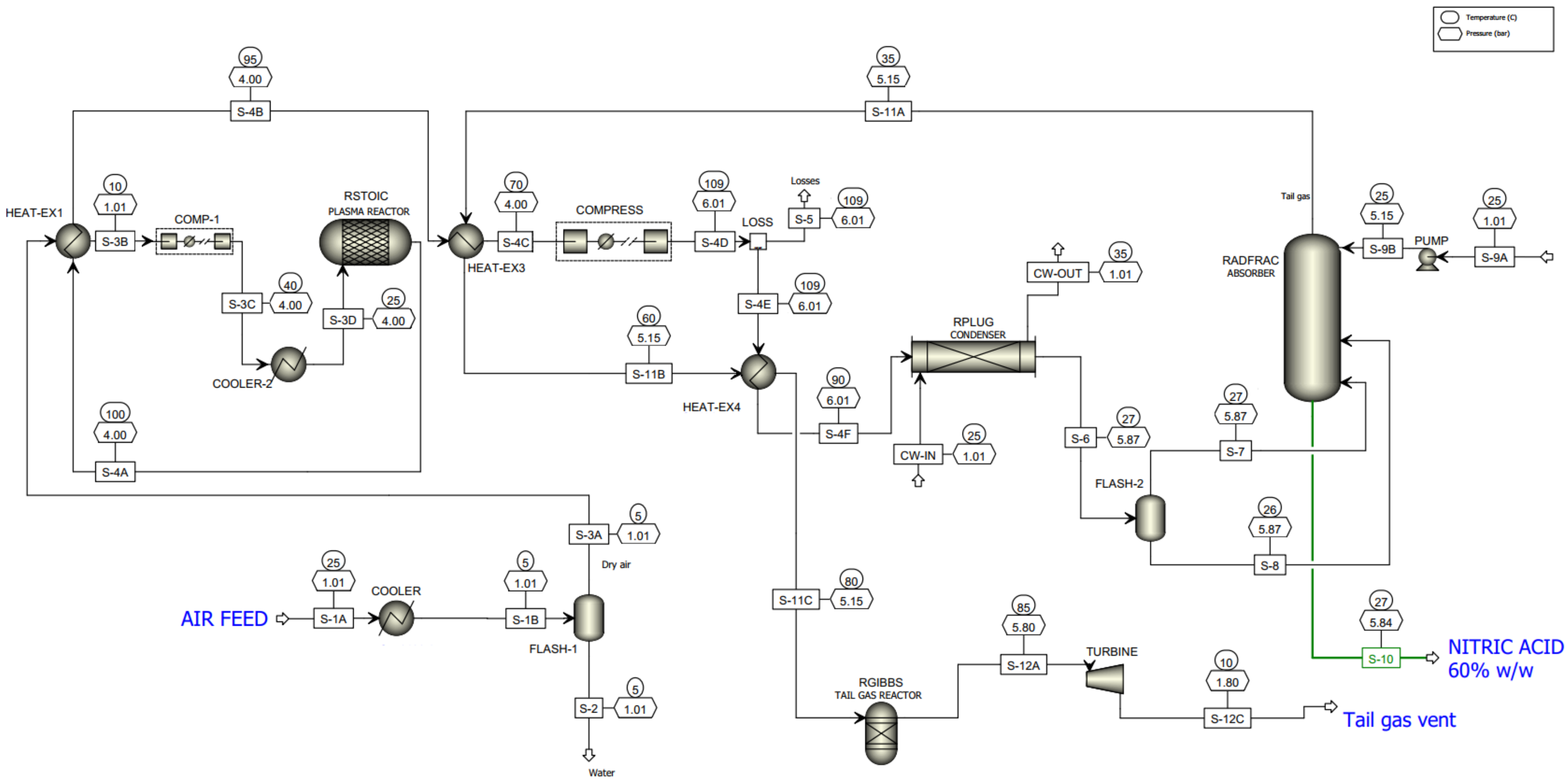

Figure S2. Nitric acid production plant using an elevated pressure rotating gliding arc plasma reactor with 4:1 N_2:O_2 ratio

Table S1. Details of the solar farm facilities presented in the photographs in Figure 3 of the main text

Figure panel	Component shown	Full description
(a)	Roseworthy Campus Solar Farm overview	Overview of the Roseworthy Campus Solar Farm, featuring the 1.2 MW ground-mounted solar photovoltaic (PV) array, Tesla Battery Energy Storage System (BESS), main PV distribution board, and the 11 kV high-voltage Sub 203 transformer kiosk.
(b)	PV distribution board	The 400 V / 2500 A PV distribution board, serving as the central AC aggregation point for the 14 × 75 kW SMA string inverters. The weather-proof enclosure integrates the system's protection circuit breakers, billing meters, and microgrid control relays.
(c)	11 kV step-up transformer	Close-up of the outdoor-rated 11 kV Sub 203 three-phase high-voltage step-up transformer, which steps up 400 V power from the PV distribution board to 11,000 V for efficient distribution across the campus network.
(d)	Tesla BESS Powerpack unit	Tesla commercial-scale Battery Energy Storage System (BESS) Powerpack unit, providing localised grid support, peak demand management, and energy arbitrage capabilities, with a storage capacity of 290 kW / 1160 kWh.
(e)	Solar PV array	Segment of the approximately 3,200 monocrystalline solar PV panels, based on JA Solar 380 Wp modules, securely mounted on an engineered fixed-tilt steel racking system optimised at a 20° tilt angle and facing true north.

Table S2. PV Array Operational and Technical Specifications

Specification Parameter	Sub-array #1	Sub-array #2	Combined Total
**PV Module Type / Model**	Monocrystalline Si (JA Solar JAM72S01-380/PR/1500V)	Monocrystalline Si (JA Solar JAM72S01-380/PR/1500V)	—
**Unit Module Rating**	380 Wp	380 Wp	—
**Series / Parallel Configuration**	18 modules/string × 156 strings	18 modules/string × 20 strings	176 strings total
**Total PV Modules**	2,808 modules	360 modules	**3168 modules**
**Nominal Power Capacity (STC)**	1,067 kWp	137 kWp	**1204 kWp**
**Power Capacity at 50°C**	966 kWp	124 kWp	**1090 kWp**
**MPP Voltage (Umpp) at 50°C**	645 V	645 V	**645 V**
**MPP Current (I{mpp) at 50°C**	1,497 A	192 A	**1689 A**
**Total Module Area**	—	—	**6153 m^2**
**Active Cell Area**	—	—	**5572 m^2**
